# Supplementary material for: Construction of a mammalian embryo model from stem cells organized by a morphogen signalling centre
Source: Nat Commun. 2021 Jun 2;12:3277. doi: 10.1038/s41467-021-23653-4 (PMC8172561; doi:10.1038/s41467-021-23653-4)
Supplement: Supplementary file 1 — Supplementary information [file 41467_2021_23653_MOESM1_ESM.pdf]

Construction of a Mammalian Embryo Model from Stem Cells Organized by a Morphogen Signalling Centre

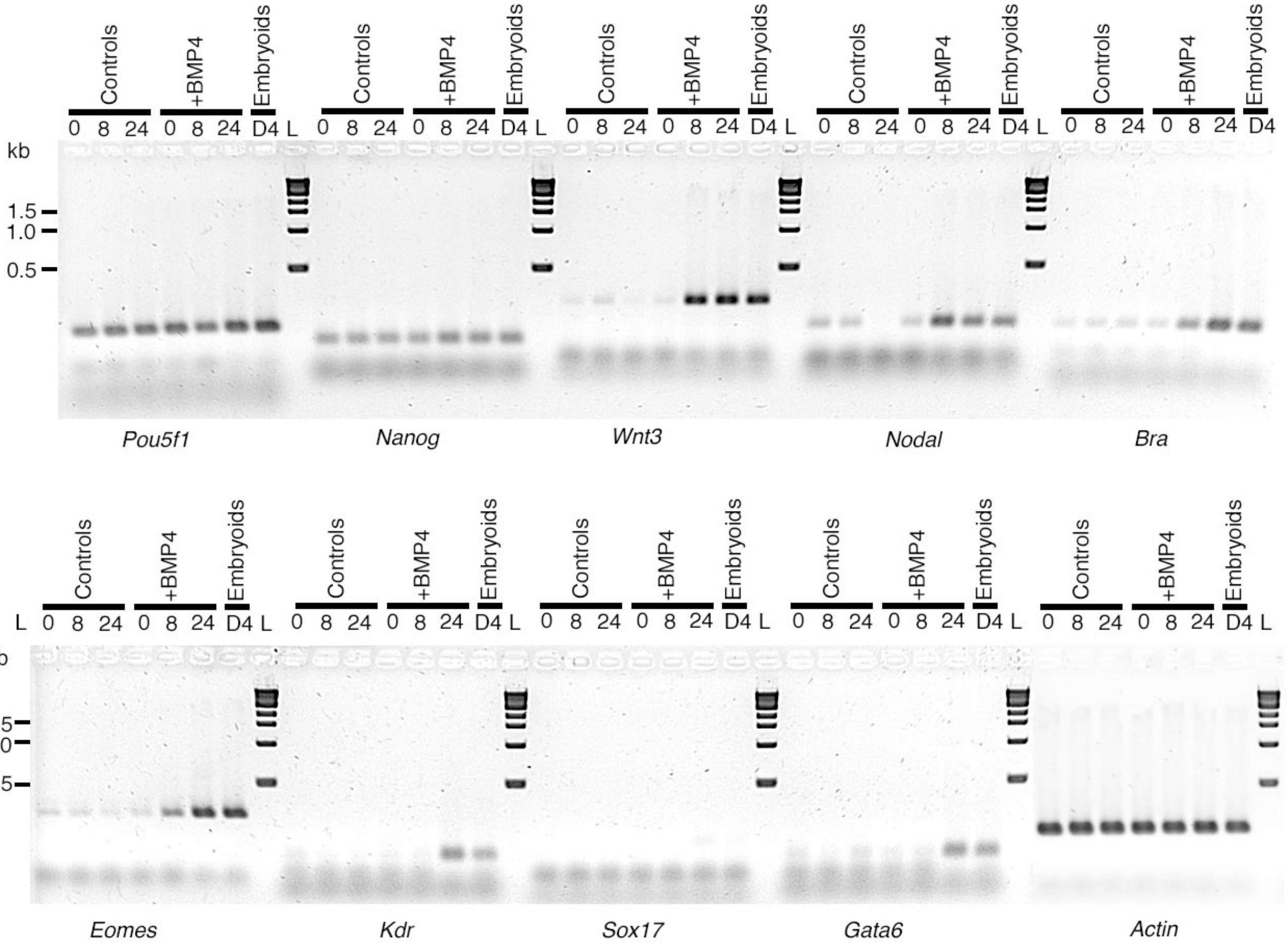

	Control 0h	Control 8h	Control 24h	+ BMP4 0h	+ BMP4 8h	+ BMP4 24h	Embryoid D4
Pou5f1	+++	+++	+++	+++	+++	+++	+++
Nanog	++	++	++	++	++	++	++
Wnt3	+	+	+	+	+++	+++	+++
Nodal	+	+	-	+	+++	++	++
T	+	+	+	+	++	+++	+++
Eomes	+	+	+	+	++	+++	+++
Kdr	-	-	-	-	-	++	++
Sox17	-	-	-	-	-	+	+
Gata6	-	-	+	-	-	++	++
Actin	+++	+++	+++	+++	+++	+++	+++

Supplementary Fig. 1: Characterization of naive and instructed aggregates

Top: RT-PCR for the pluripotency markers *Pou5f1* and *Nanog*, the morphogens *Wnt3* and *Nodal* and their downstream targets: *Bra/T*, *Eomes*, *Kdr*, *Sox17* and for the primitive endoderm marker *Gata6*. *Actin* is used as a control.

Developmental stages for naive aggregates (Controls) and instructed aggregates (+BMP4) are indicated at the top of the figure: for Controls: 0h at D2.66; 8h: at D3, 24h at D3.66. Instructed aggregate: 0h at D2.66, 8h: 8 hours of incubation with BMP4 protein (10 µg/ml) - D3 and 24h at D3.66. Controls and instructed aggregates were grown independently until D4. Embryoids derived from merging at D3 of naive aggregates with instructed aggregates (incubated 8 hours with BMP4 protein) analysed at D4.

Number of aggregates/embryoids pooled per stage: controls and instructed aggregate 0h, N=288; instructed aggregate (8h + BMP4): N=192; 24hs N=192; Controls 8h: N=96; 24h: N=96; Embryoids D4, N=96.

Bottom: Semi-quantitative interpretation of the RT-PCR.
Pluripotency markers were expressed without change for all stages and conditions analysed. Morphogens *Wnt3* and *Nodal* were weakly expressed at D2.66, D3 and D4 in controls and their expression was strongly induced by BMP4. Downstream targets of these morphogens were either weakly expressed or not expressed at D2.66 and D3 in both naive and instructed aggregates but their expression was immediately induced (*Bra/T*, *Eomes*) after 8h of incubation with BMP4 or induced after a delay (*Kdr*, *Sox17*). Finally, the primitive endoderm marker *Gata6* was not expressed until D4 and its transcripts were barely detectable in Controls at that stage while its expression was induced in the instructed aggregates. Expression data for D4 embryoids were similar to the expression data of the instructed aggregates at that stage. The uncropped images of the gels are presented in Source data file – Supplementary Fig. 1.

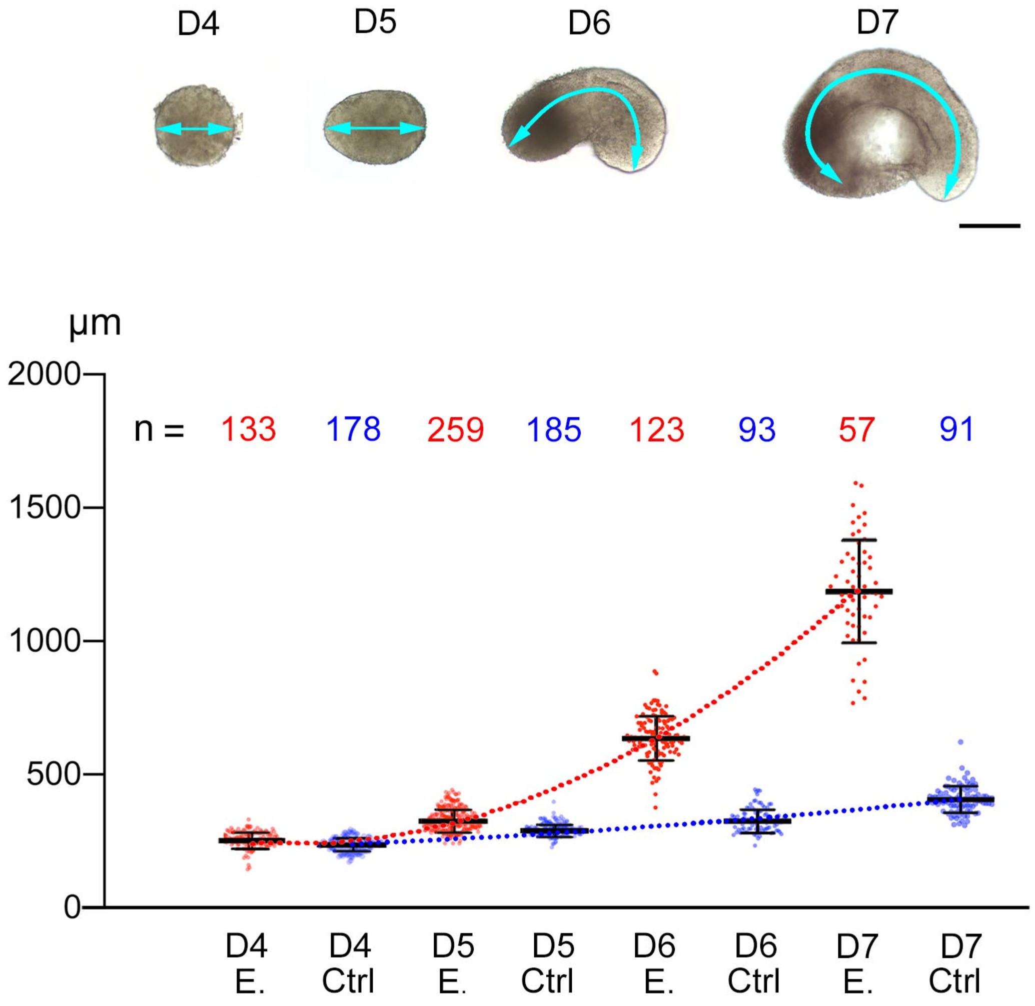

Supplementary Fig. 2: **Elongation of embryoids from D4 to D7**

(Top) Lateral view of typical embryoids at D4, D5, D6 and D7. Blue double-headed arrows mark their AP axis. Scale bar: 200µm. (Bottom) Graph indicating the mean of length (in µm) of embryoids (E., red dots) along their AP axis or the diameter of control aggregates (Ctrl, blue dots) from D4 to D7. Error bars, means ± standard deviation, dots indicate individual measurements and n is the number of embryoids or of control aggregates measured. The equations for the growth curve are $y=119x^2 - 286.7x + 415.35$ ($R^2=1$) for the growth of embryoids and $y=7.425x^2 + 17.805x + 210.97$ ($R^2=0.9876$) for the growth of control aggregates. Scale bar: 100µm.

Numerical data used for this graph are presented in Source data file – Supplementary Fig. 2.

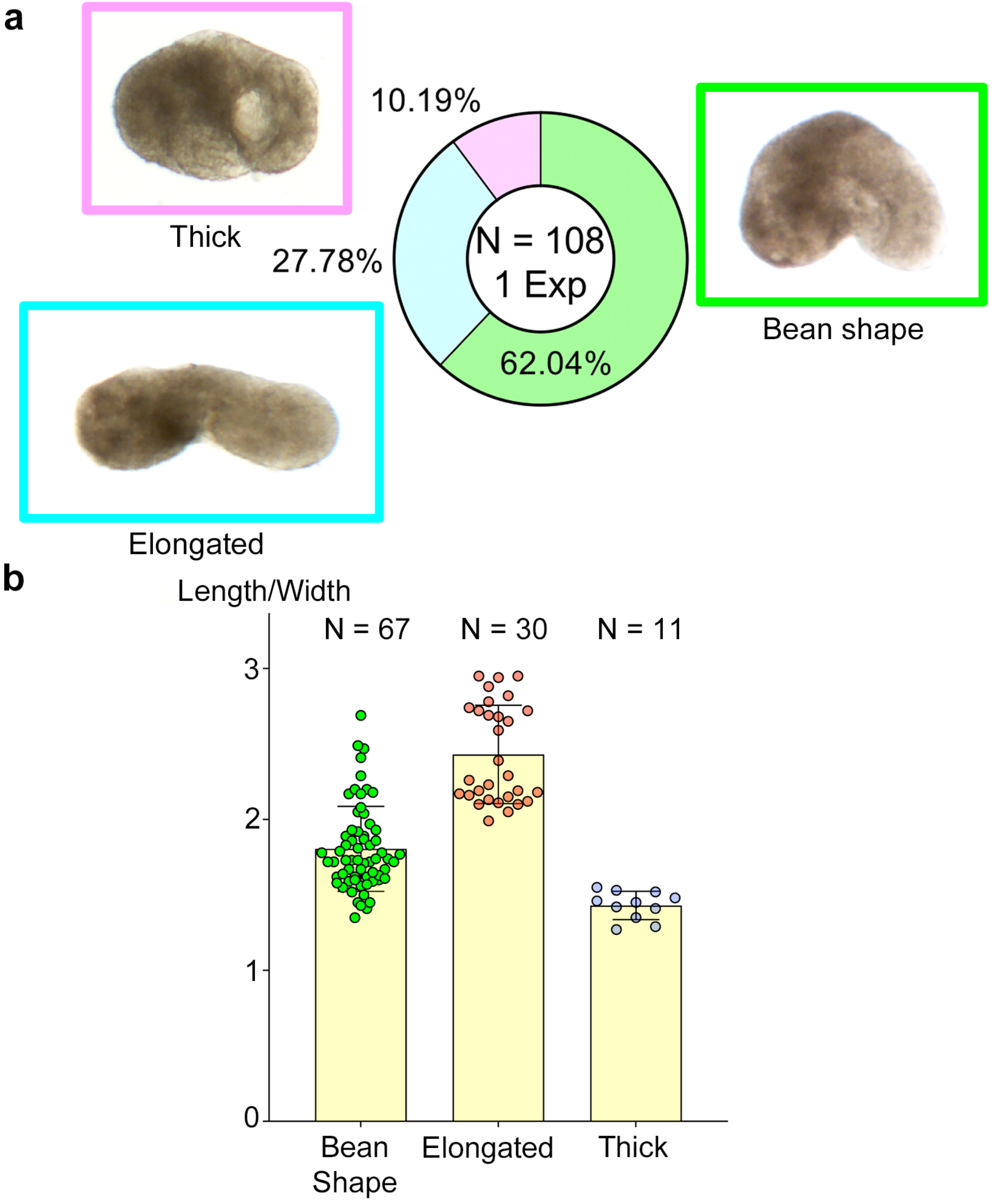

Supplementary Fig. 3: Morphological variability of embryoids at D7

Embryoids have been measured for their extension in length and width and their compaction or elongation analysed based on their length/width ratio for three classes of embryoids: bean shape, elongated and thick. (**a**) Characterisation of the different classes. The percentage of embryoids in each group was presented in a donut graph, with an associated picture for a representative embryoid (same colour codes for the box surrounding the picture and the fraction of the donuts graph representing the percentage). N = 108 embryoids, 1 Experiment. (**b**) Graph representing the distribution of the length/width ratio of analysed embryoids. N indicates the number of embryoids in each class. Error bars are means ± standard deviation. Scale bar: 100 µm.

Numerical data used for this graph and statistics are presented in Source data file Supplementary Figure 3.

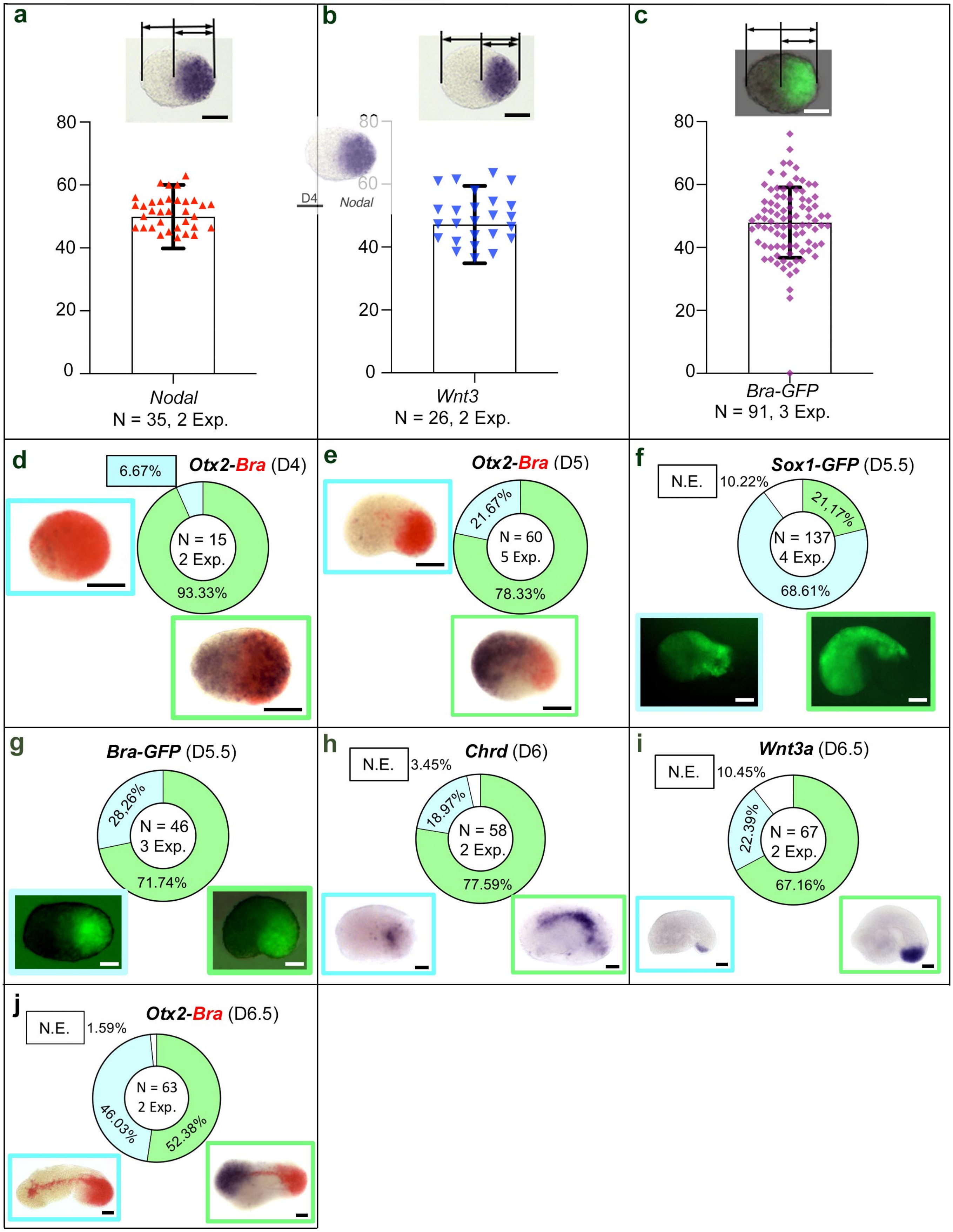
a
80
60
40
20
0
Nodal
N = 35, 2 Exp.
b
D4
Nodal
Wnt3
N = 26, 2 Exp.
c
Bra-GFP
N = 91, 3 Exp.
d
6.67%
Otx2-Bra (D4)
N = 15
2 Exp.
93.33%
e
Otx2-Bra (D5)
21.67%
N = 60
5 Exp.
78.33%
f
N.E.
10.22%
Sox1-GFP (D5.5)
21,17%
N = 137
4 Exp.
68.61%
g
Bra-GFP (D5.5)
28,26%
N = 46
3 Exp.
71.74%
h
N.E.
3.45%
Chrd (D6)
18.97%
N = 58
2 Exp.
77.59%
i
N.E.
10.45%
Wnt3a (D6.5)
22.39%
N = 67
2 Exp.
67.16%
j
N.E.
1.59%
Otx2-Bra (D6.5)
N = 63
2 Exp.
46.03%
52.38%

Supplementary Fig. 4: Variability of the different gene expression patterns described in Fig. 1.

For each experiment, the numbers of embryoids (N) and of independent experiments (Exp.) were indicated. (**a-c**) For *Nodal* (**a**), *Wnt3* (**b**) and *Bra-GFP* (**c**) the variability in expression patterns has been evaluated by measuring the posterior-anterior extent of their expression expressed as a percentage of the embryo length. The graph indicates the mean for each experiment with error bars indicating standard deviation; dots indicate individual measurement. Numerical data are provided in Source Data file Supplementary Figure 4. (**d-j**) Variability in expression patterns within the population of embryoids was analysed for each gene or combination of genes (**d**) *Otx2-Bra* at D4, (**e**): *Otx2-Bra* at D5, (**f**) *Sox1-GFP* at D5.5, (**g**) *Bra-GFP* at D5.5, (**h)** *Chrd* at D6, (**i**) *Wnt3a* at D6.5 and (**j**) *Otx2-Bra* at D6.5. The population of embryoids has been distributed in groups showing significant differences in expression patterns from one another. The percentage of embryoids in each group is presented in a donuts graph, with an associated picture for a representative embryoid (same colour codes for the box surrounding the picture and the fraction of the donuts graph representing the percentage). N.E.: Not expressed. The group of embryoids that did not express the analysed gene(s) is indicated in white. The name of the gene(s) analysed and the developmental stages of the embryoids are indicated on top in each panel. Scale bars: 100 µm.

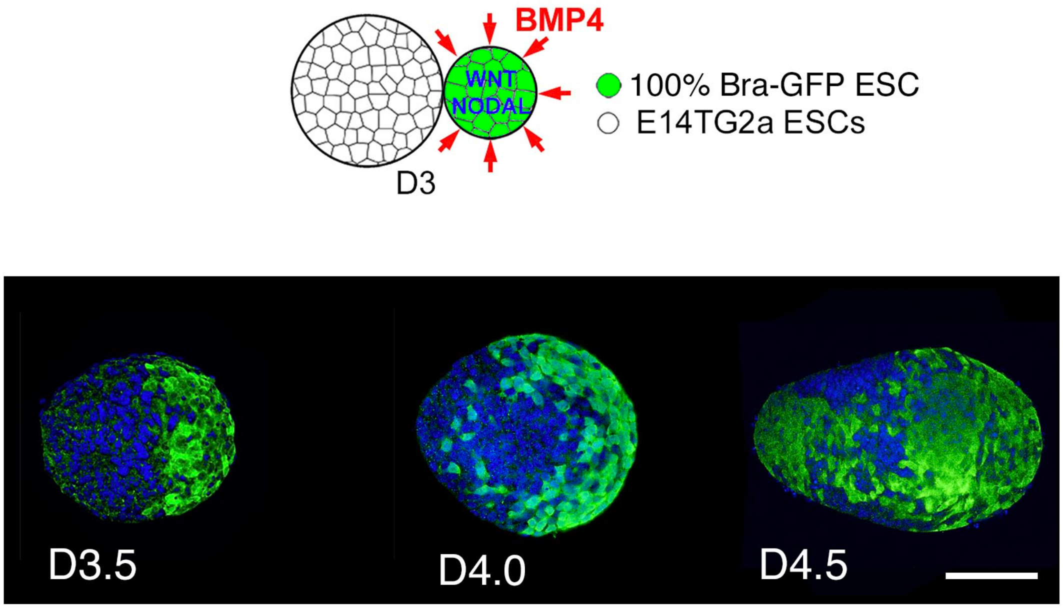

Supplementary Fig. 5: Position over time of mesodermal cells in embryoids

Top: Drawing summarizing the experimental strategy. A naive, unlabelled ESC aggregate was fused to a BMP4 instructed aggregate made of *Bra-GFP* cells. The activity of NODAL and WNT induced by BMP4 in the instructed aggregate resulted in the expression of the mesodermal marker *Bra-GFP*.

Bottom: Images of embryoids at D3.5, D4 and D4.5 showing all nuclei (Dapi, blue) and mesodermal cells expressing *Bra-GFP* (green). Note that the organisation of *Bra* expressing cells is not epithelial but mesenchymal. Number of embryoids analysed with similar result: D3.5 (N=12, 2 experiments), D4.0 (N=11, 2 experiments) and D4.5 (N=10, 2 experiments). The expression level of D3.5, 12 h after merging, was low and a longer exposition time resulted in high background (small green speckles). In order to better visualize *Bra-GFP* expressing cells, this background has been diminished using the function noise/despeckle feature of Photoshop (with radius = 2; threshold = 0). Scale bar: 100µm.

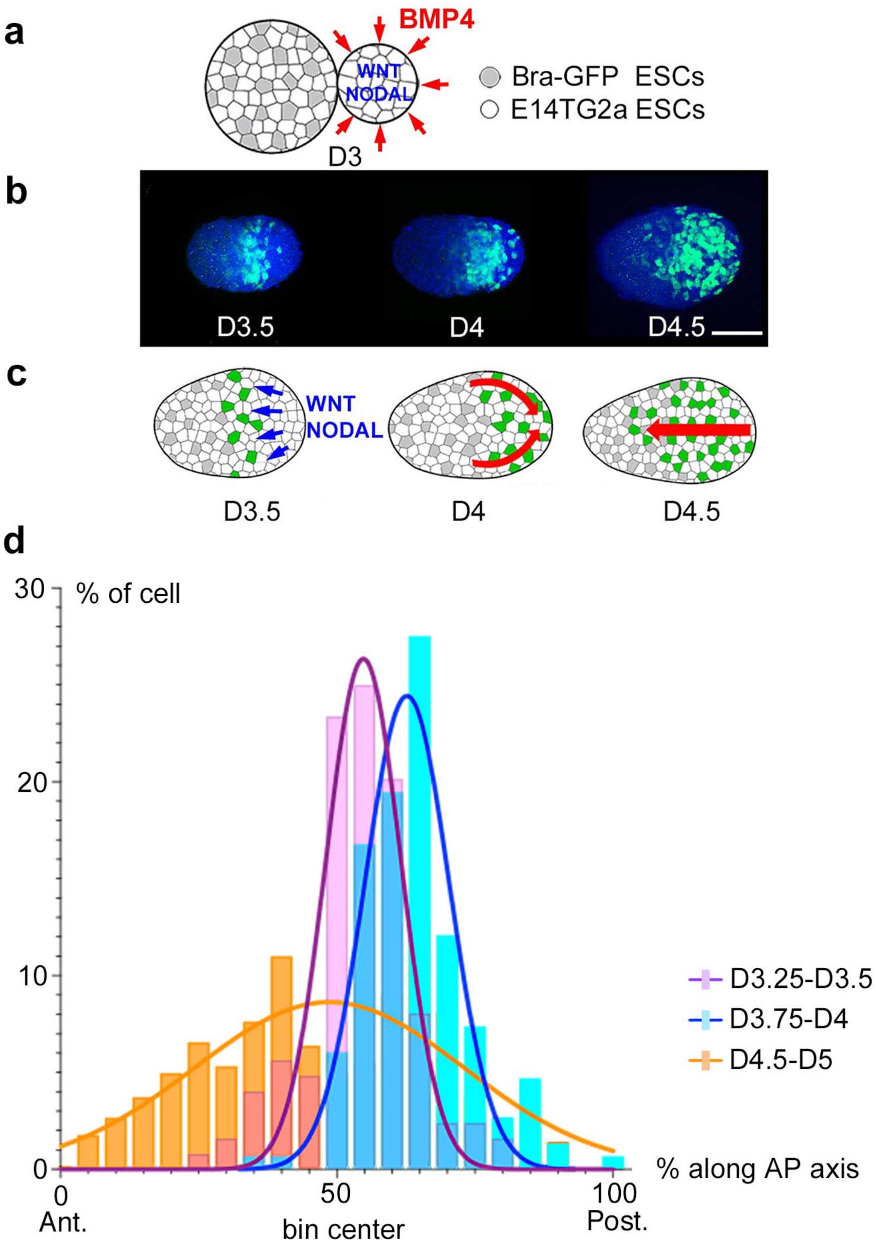

e

	D3.25-D3.5	D3.75-D4	D4.5-D5
Total number of values	124	149	564
Number of excluded values	0	0	0
Number of binned values	124	149	564
Minimum	24,6298789	37,2239748	2,3316062
25% Percentile	51,00052519	57,61611236	32,42935442
Median	54,15280867	64,3533123	46,39916641
75% Percentile	59,81707035	69,07544808	65,15343964
Maximum	81,79287319	97,7917981	92,05077508
Mean	54,22060135	64,50925089	47,61395898
Std. Deviation	9,714681086	9,663230157	20,55943732
Std. Error of Mean	0,872404115	0,79164266	0,865708482
Lower 95% CI of mean	52,4937309	62,94486803	45,91354604
Upper 95% CI of mean	55,9474718	66,07363376	49,31437191

Supplementary Fig. 6: Frequency distribution over time of mesodermal cells induced in territories adjacent to the organizing centre

(**a-c**) Summary of the experiment described in **Fig. 2**, with the naive domain of the embryoids containing 10% *Bra-GFP* ESC and the signalling centre made entirely of unlabelled cells. Scale bar: 100 µm. Number of embryoids analysed in (**b**): D3.5, N=21; D4, N=18 and D4.5, N=12 in two independent experiments (**d**) Frequency distribution with a bin width of 5, of *Bra-GFP* expressing cells for embryoids at D3.25-D3.5, D3.75-D4 and D4.5-D5 stages along the anterior (Ant.) to posterior (Post.) axis. Curves represent the Gaussian fit of histogram of frequency distribution of *Bra-GFP* expressing cells. Measurements have been done in two independent experiments for a total of 21, 18 and 12 embryoids at D3.25-D3.5, D3.75-D4 and D4.5-D5 respectively. (**e**) Statistics of the graph in (**d**). Scale bar: 100µm. Numerical data for this graph are presented in a Source Data file Supplementary Figure 6.

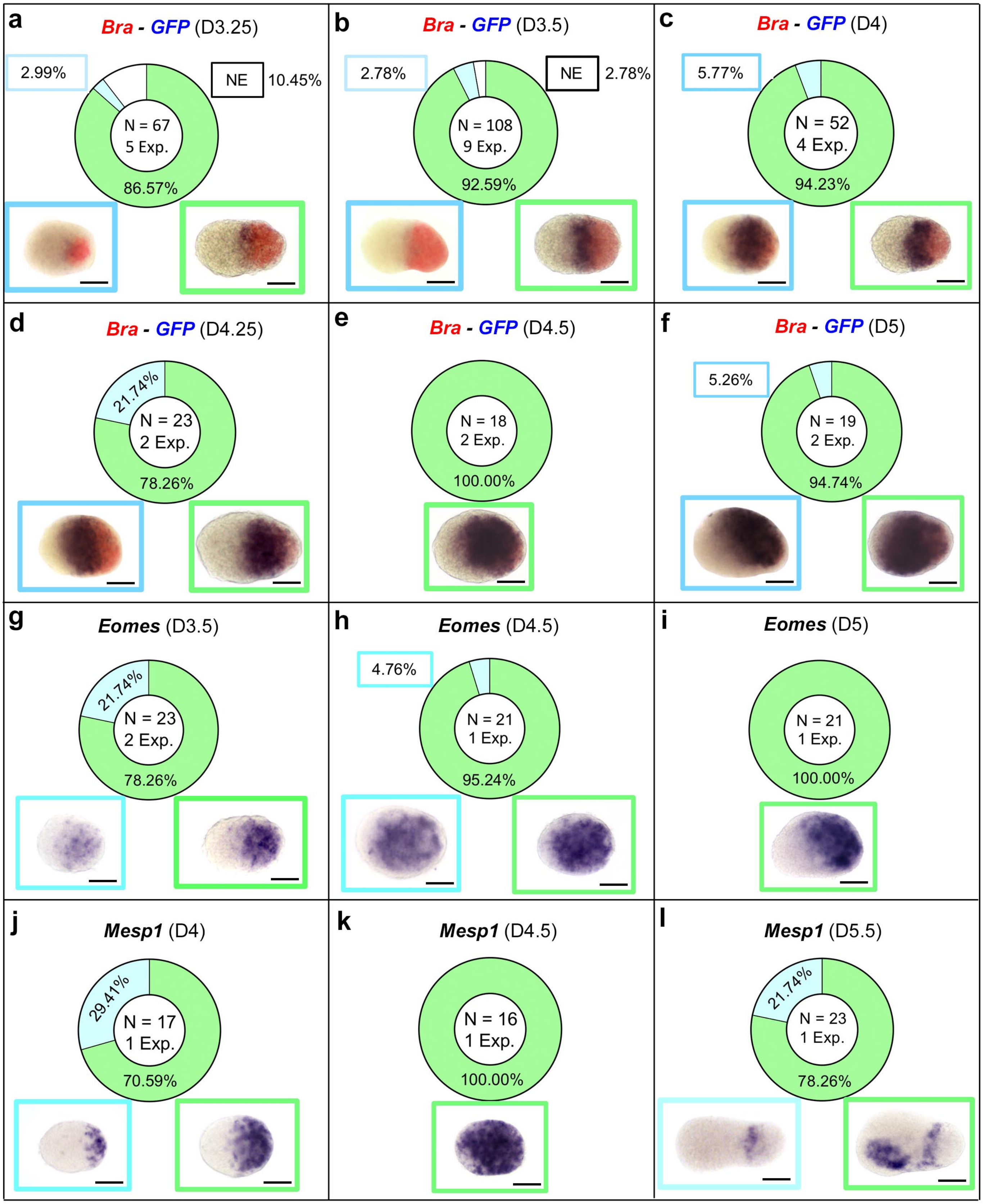

a
Bra - GFP (D3.25)
2.99%
NE
10.45%
N = 67
5 Exp.
86.57%
b
Bra - GFP (D3.5)
2.78%
NE
2.78%
N = 108
9 Exp.
92.59%
c
Bra - GFP (D4)
5.77%
N = 52
4 Exp.
94.23%
d
Bra - GFP (D4.25)
21.74%
N = 23
2 Exp.
78.26%
e
Bra - GFP (D4.5)
N = 18
2 Exp.
100.00%
f
Bra - GFP (D5)
5.26%
N = 19
2 Exp.
94.74%
g
Eomes (D3.5)
21.74%
N = 23
2 Exp.
78.26%
h
Eomes (D4.5)
4.76%
N = 21
1 Exp.
95.24%
i
Eomes (D5)
N = 21
1 Exp.
100.00%
j
Mesp1 (D4)
29.41%
N = 17
1 Exp.
70.59%
k
Mesp1 (D4.5)
N = 16
1 Exp.
100.00%
l
Mesp1 (D5.5)
21.74%
N = 23
1 Exp.
78.26%

Supplementary Fig. 7: Variability of the different gene expression patterns described in Fig. 2g-i.

(**a-l**) Variability in expression patterns for (**a**) *Bra* and *GFP* at D3.25, (**b**) *Bra* and *GFP* at D3.5, (**c**) *Bra* and *GFP* at D4, (**d**) *Bra* and *GFP* at D4.25, (**e**) *Bra* and *GFP* at D4.5, (**f**) *Bra* and *GFP* at D5, (**g**) *Eomes* at D3.5, (**h**) *Eomes* at D4.5, (**i**) *Eomes* at D5, (**j**) *Mesp1* at D4, (**k**) *Mesp1* at D4.5 and (**l**) *Mesp1* at D5.5. For each experiment, the numbers of embryoids (N) and of independent experiments (Exp.) were indicated. To evaluate the variability in expression patterns in the population of embryoids analysed, for each gene expression or combination of genes expression, the population of embryoids have been distributed in groups showing significantly different expression patterns from one to the other. The percentage of embryoids in each group is presented in a donut graph with the associated picture of a representative embryoid with the same colour code for the box surrounding the picture and the fraction of the donuts graph representing the percentage. N.E.: Not expressed. The group of embryoids that did not express the analysed gene(s) is indicated in white. The name of the gene(s) analysed and the developmental stages of the embryoids are indicated at the top of each panel. Scale bars: 100µm.

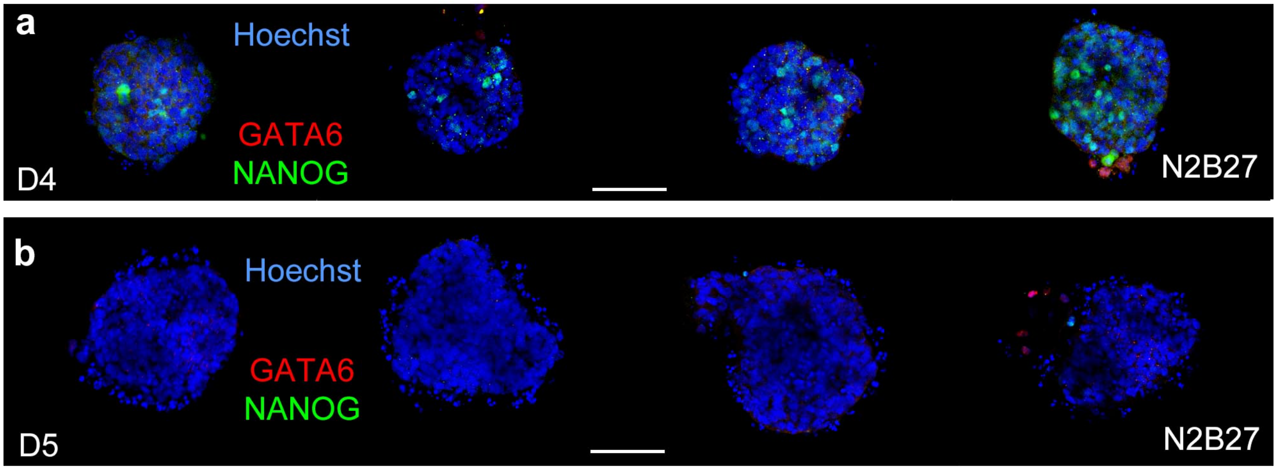

Supplementary Fig. 8: Expression of NANOG and GATA6 in naive ESC aggregates.
(**a-b**) Immunolabelling for GATA6 (red) and NANOG (green) of four naive ESC aggregates cultured in N2B27 medium at D4 (**a**) and at D5 (**b**). Number of aggregates analysed: D4, N=16 and D5, N=12 in 2 independent experiments. Scale bars: 100µm.

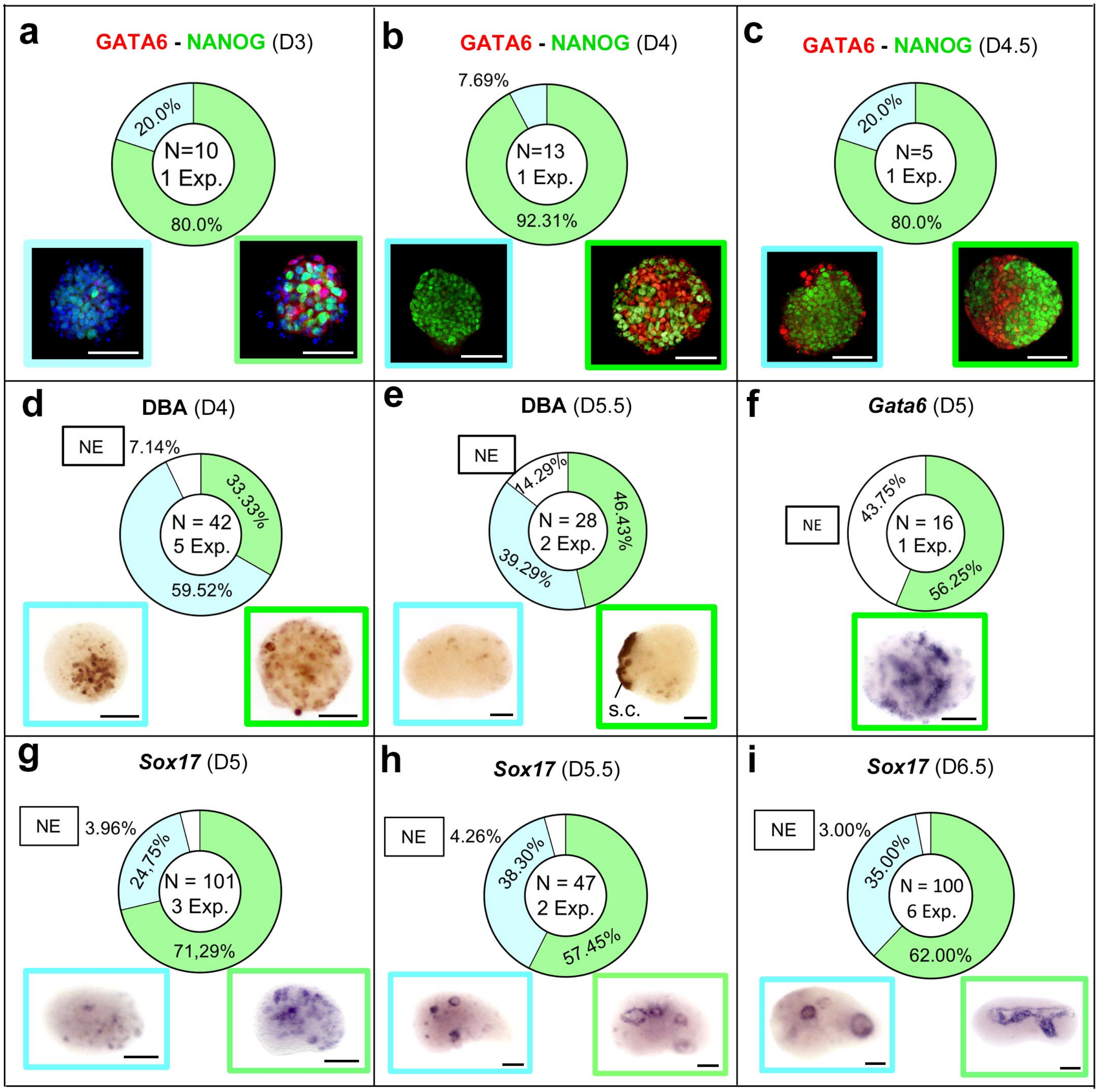

Supplementary Fig. 9: Variability of the different gene expression patterns described in Fig. 3a-l. (**a-i**)Variability in expression patterns for (**a**) GATA6 and NANOG at D3, (**b**) GATA6 and NANOG at D4, (**c**) GATA6 and NANOG at D4.5, (**d**) DBA labelling at D4, (**e**) DBA labelling at D5.5, (**f**) *Gata6* at D5, (**g)** *Sox17* at D5, (**h)** *Sox17* at D5.5 and (**i)** *Sox17* at D6.5. For each experiment performed the numbers of embryoids (N) and of independent experiments (Exp.) were indicated. To evaluate the variability in expression patterns in the population of embryoids analysed, for each gene expression or combination of genes expression, the population of embryoids have been distributed in groups showing various expression patterns from each other. The

percentage of embryoids in each group was presented in a donuts graph with the associated picture of a representative embryoid with the same colour code for the box surrounding the picture and the fraction of the donuts graph representing the percentage. N.E.: Not expressed. The group of embryoids that did not express the analysed gene(s) is indicated in white. s.c: superficial clump of ve cells. Names of the gene(s) analysed or treatment (DBA labelling) and developmental stages of the embryoids are indicated at the top of each panel. Scale bars: 100µm.

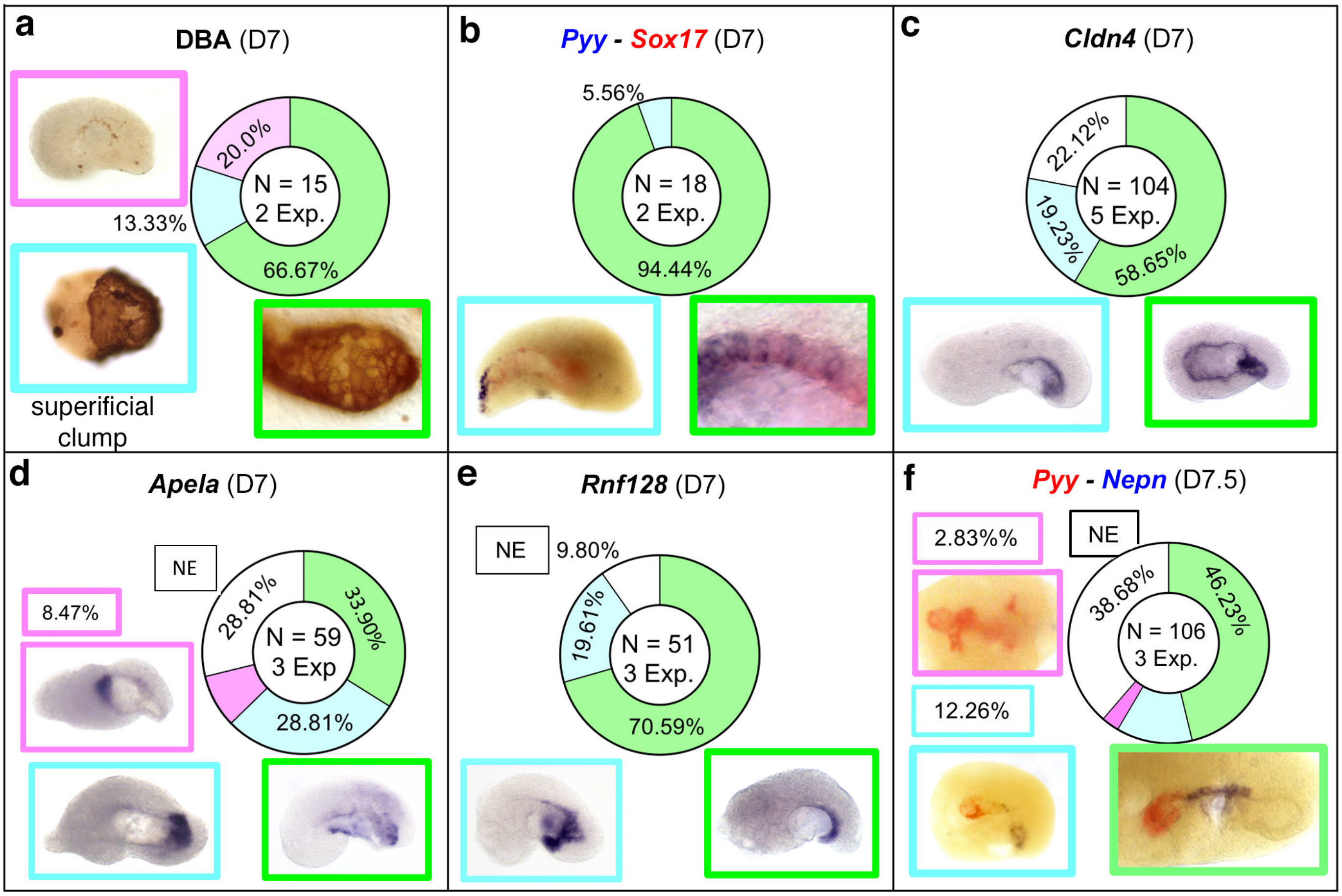

Supplementary Fig. 10: Variability of the different gene expression patterns described in Fig. 3m-v. (**a-f**) Variability in expression patterns for (**a**) DBA labelling at D7, (**b**) *Pyy* and *Sox17* at D7; (**c**) *Cldn4* at D7, (**d**) *Apela* at D7, (**e**) *Rnf128* at D7 and (**f**) *Pyy* and *Nepn* at D7.5. For each experiment presented in **Fig. 3m-v**, numbers of embryoids (N) and of independent experiments (Exp.) are indicated. To evaluate the variability in expression patterns in the population of embryoids analysed, for each gene expression or combination of genes expression, the population of embryoids has been distributed in groups showing significant different expression patterns from each other. The percentage of embryoids in each group was presented as a donut graph with the associated picture of a representative embryoid with the same colour code for the box surrounding the picture and the fraction of the donuts graph representing the percentage. N.E.: The group of embryoids that did not express the analysed gene(s) is indicated in white. Names of the gene(s) analysed or treatment (DBA labelling) and developmental stages of the embryoids are indicated at the top of each panel. Scale bars: 100µm.

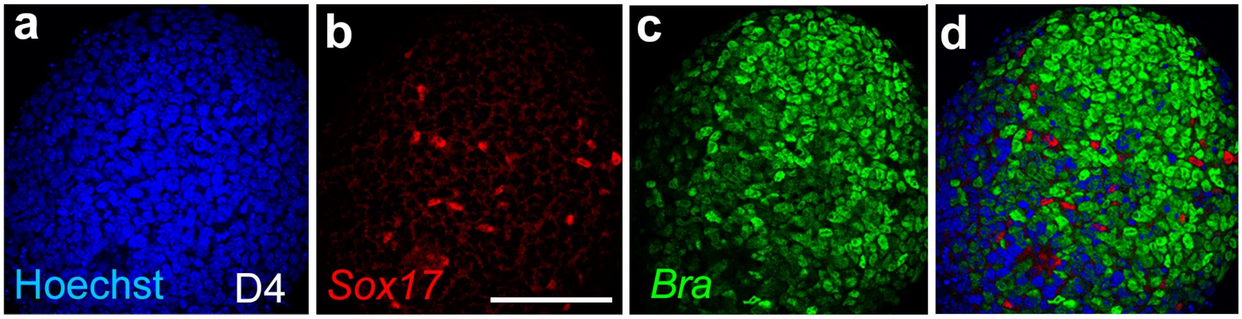

Supplementary Fig. 11: Endoderm and mesoderm do not derive from a pool of mesendoderm progenitors.

(a) Embryoid at D4 labelled with Hoechst 33342 (labelling nuclei). (**b-c**) Immunolabelling of the same embryoid for *Sox17* (red, **b**) *Bra* (green, **c**). (**d**) Merge of panels **a-c** showing that none of the D4 (gastrulating) embryoids coexpressed the endodermal marker *Sox17* and the mesodermal marker *Bra.* Number of embryoids analysed with similar result: N= 10/10 in 2 experiments. Scale bar: 100µm.

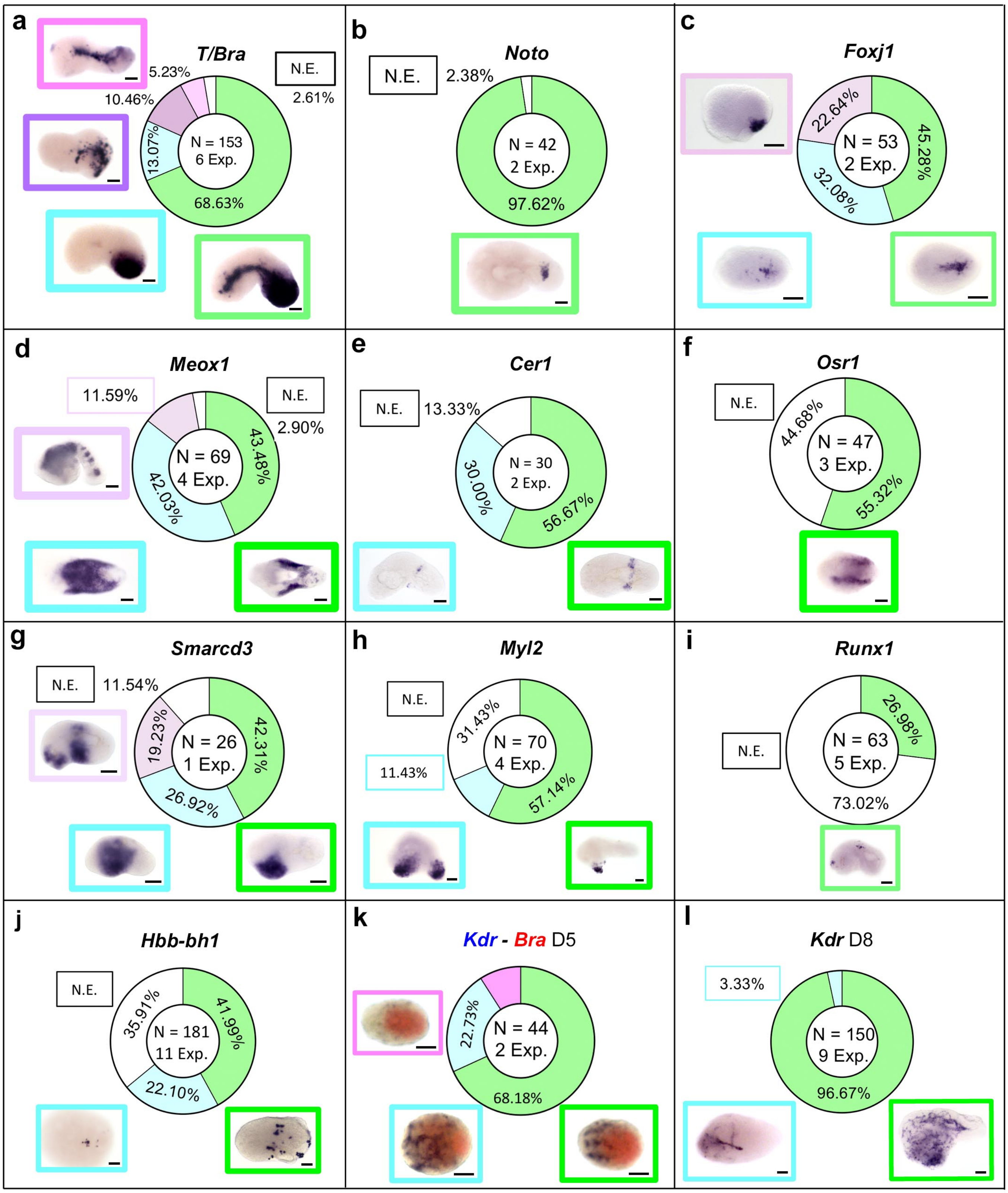

Supplementary Fig. 12: Variability of the different gene expression patterns described in Fig. 4.

(**a-l**) Variability in expression patterns for (**a**) *T/Bra* at D7, (**b**) *Noto* at D7, (**c**) *Foxj1* at D5.5, (**d**) *Meox1* at D7, (**e**) *Cer1* at D7, (**f**) *Osr1* at D7, (**g**) *Smarcd3* at D6, (**h**) *Myl2* at D7.5, (**i**) *Runx1* at D6, (**j**) *Hbb-bh1* at D8, (**k**)

Kdr and *Bra* at D5 and (**I**) *Kdr* at D8. For each experiment presented in **Fig. 4**, the numbers of embryoids (N) and the number of independent experiments (Exp.) are indicated. To document the variability in expression patterns within each population of embryoids analysed, the percentage of embryoids with each of the different expression patterns are shown in a donut graph with the associated picture of a representative embryoid with the same colour code for the box surrounding the picture and the fraction of the donuts graph representing the percentage. N.E.: The group of embryoids that did not express the analysed gene(s) is indicated in white. Names of the gene(s) analysed and developmental stages of the embryoids were indicated at the top of each panel. Scale bars: 100µm.

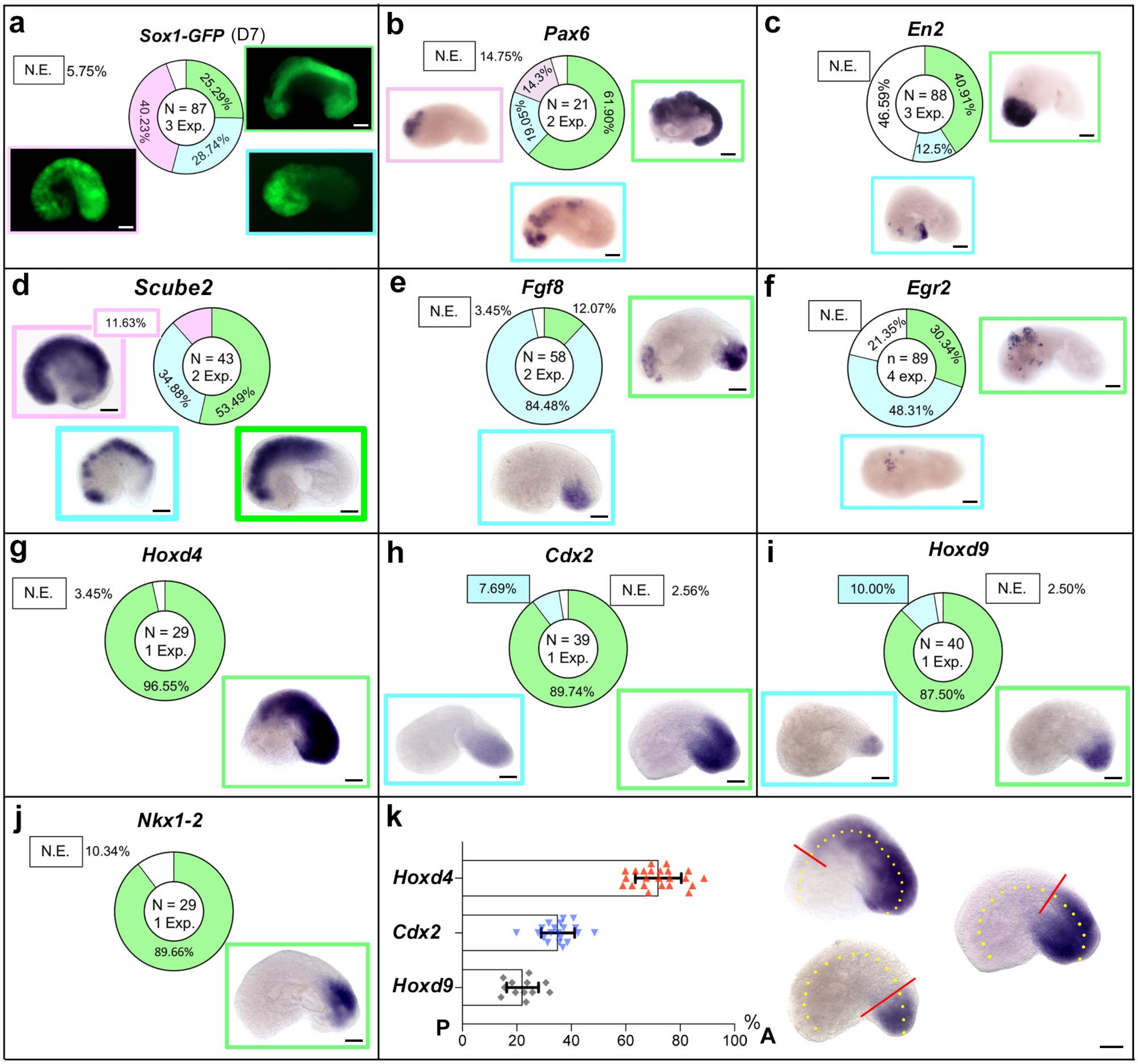

Supplementary Fig. 13: Variability of the different gene expression patterns described in Fig. 5

(**a-j**) Variability in expression patterns for (**a**) *Sox1-GFP* at D7, (**b**) *Pax6* at D7, (**c**) *En2* at D7, (**d**) *Scube2* at D7, (**e**) *Fgf8* at D7, (**f**) *Egr2* at D7, (**g**) *Hoxd4* at D7, (**h**) *Cdx2* at D7, (**i**) *Hoxd9* at D7 and (**j**) *Nkx1-2* at D7. For each experiment presented in **Fig. 5**, the numbers of embryoids (N) and the number of independent experiments (Exp.) are indicated. To evaluate the variability in expression patterns within the population of embryoids analysed, the percentage of embryoids with each expression pattern are shown in a donut graph with the associated picture of a representative embryoid with the same colour code for the box surrounding the picture and the fraction of the donuts graph representing the percentage. N.E.: Not expressed. The group of embryoids

that did not express the analysed gene(s) is indicated in white. (**k**) The variability in *Hoxd4*, *Cdx2* and *Hoxd9* expression was also evaluated by measuring their posterior to anterior extent. One individual embryoid for each gene was presented on the right. Yellow dots indicating the AP axis of each these embryoids while the red line indicates the anterior end of their expression pattern. Measurement of the length from the anterior most expressing cells (red line) and the tail bud was expressed in percentage of the embryo length. Error bars indicate the means ± standard deviation and the dots indicate the individual measurements (*Hoxd4*, N=23; *Cdx2*, N=20; *Hoxd9*, N=12 in one experiment). Numerical data are presented in Source Data file Supplementary Figure 13. The name of the gene analysed is indicated at the top of each panel. Embryoids are at D7. Scale bars: 100µm.

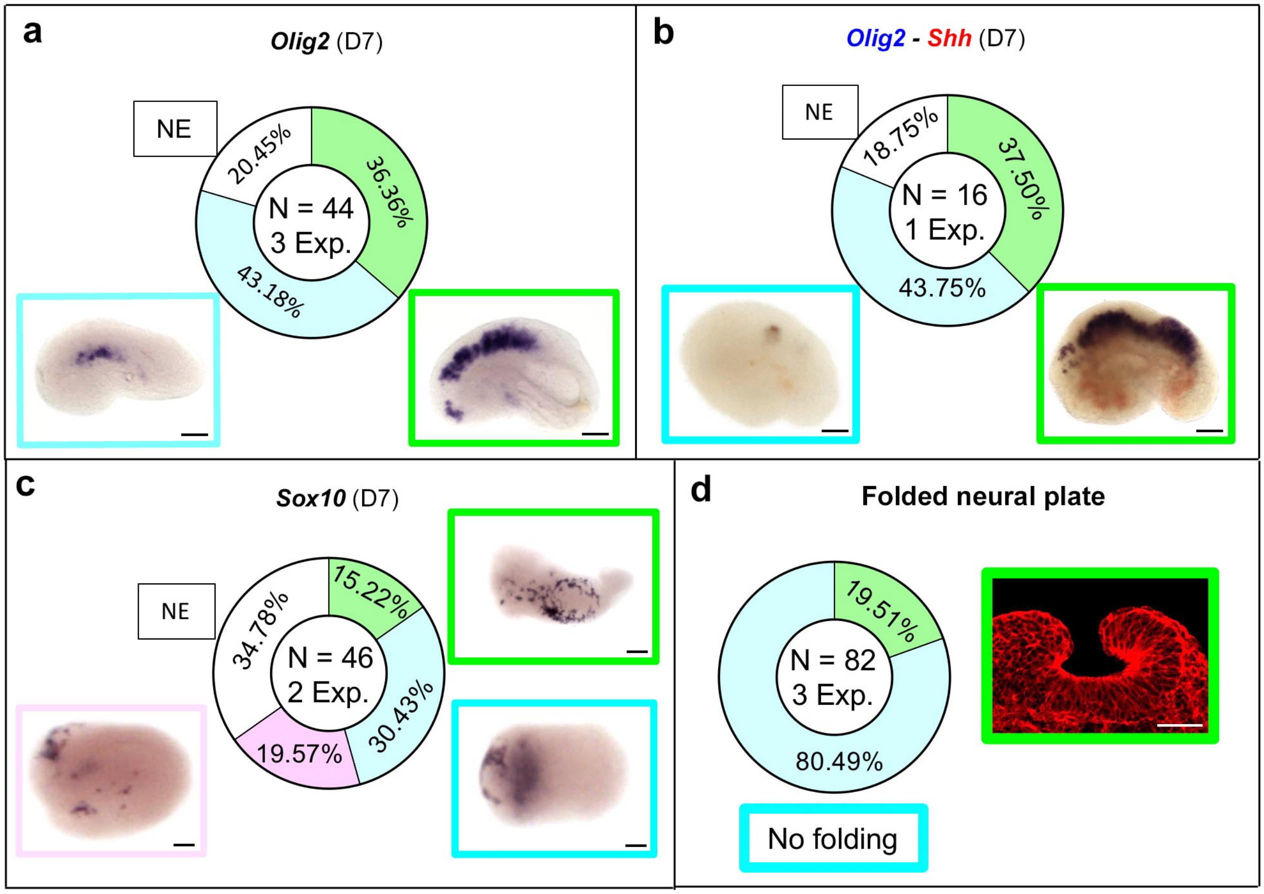

Supplementary Fig. 14: Variability of the different gene expression patterns described in Fig. 6.

(**a-c**) Variability in expression patterns for (**a**) *Olig2* at D7, (**b**) *Olig2* and *Shh* at D7, (**c**) *Sox10* at D7. For each gene whose expression was analysed, the numbers of embryoids (N) and of independent experiments (Exp.) are indicated. To evaluate the variability in expression patterns within each population of embryoids analysed, the percentage of embryoids in each group is presented in a donut graph with the associated picture of a representative embryoid with the same colour code for the box surrounding the picture and the fraction of the donuts graph representing the percentage. N.E.: Not expressed. The group of embryoids that did not express the analysed gene(s) is indicated in white. The name of the gene(s) analysed and the developmental stage of the embryoids are indicated at the top of each panel. (**d**) Frequency of folded neural plate in embryoids at D7-D9. Scale bars: 100µm.

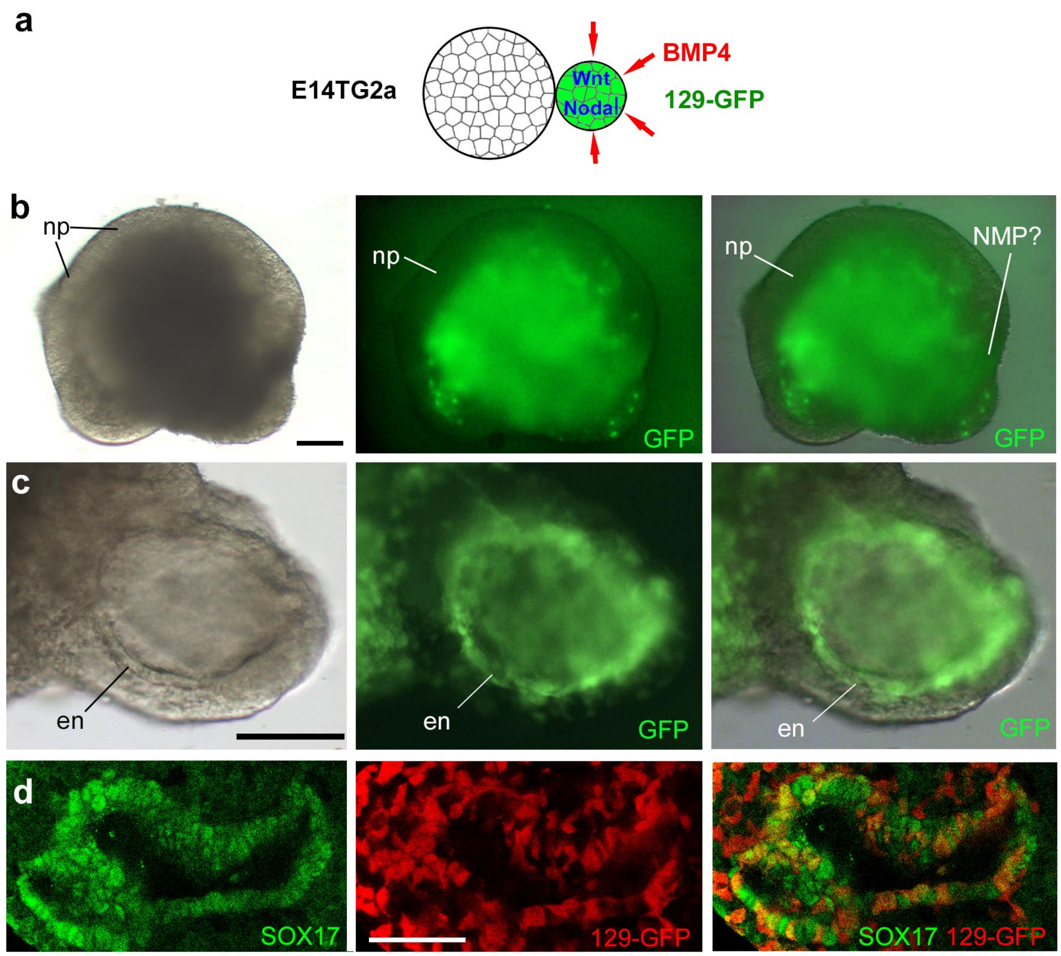

Supplementary Fig. 15: Origin of cells forming the three germ layers

(a) Schematic of the formation of an embryoid derived from an unlabelled (E14TG2 cells) naive aggregate merged with a *GFP* labelled (129-GFP cell line) signalling centre. (**b**) Brightfield, green fluorescence and merger of the tow for an embryoid at D7 made of *GFP* labelled signalling centre. Fluorescence was observed in the inner part of the embryoid (mesoderm and endoderm) while the superficial tissues (ectoderm) were unlabelled, except in the posterior territory that may represent the bipotential neuromesodermal progenitors (NMP). np: neural plate. (**c**) High magnification (brightfield, fluorescence and merged images) focusing on the endodermal throughout epithelium of a D7 embryoid made as described in (**a**) showing strong *GFP* expression in the whole epithelium. (**d**) Double immunofluorescence images for SOX17 (left), GFP (middle) and the merge image (right) of the endoderm epithelium of an embryoid at D6. The epithelium appeared mosaic with green labelled cells corresponding to Sox17(+) ve cells from the naive territory and orange/yellow cells corresponding to ve and de cells derived from the signalling centre. Number of independent experiments: 2, (**b-c**) N = 5/5; (**d**) N = 12/12. Scale bars: 100 µm (**a**, **b**); 50 µm (**d**).

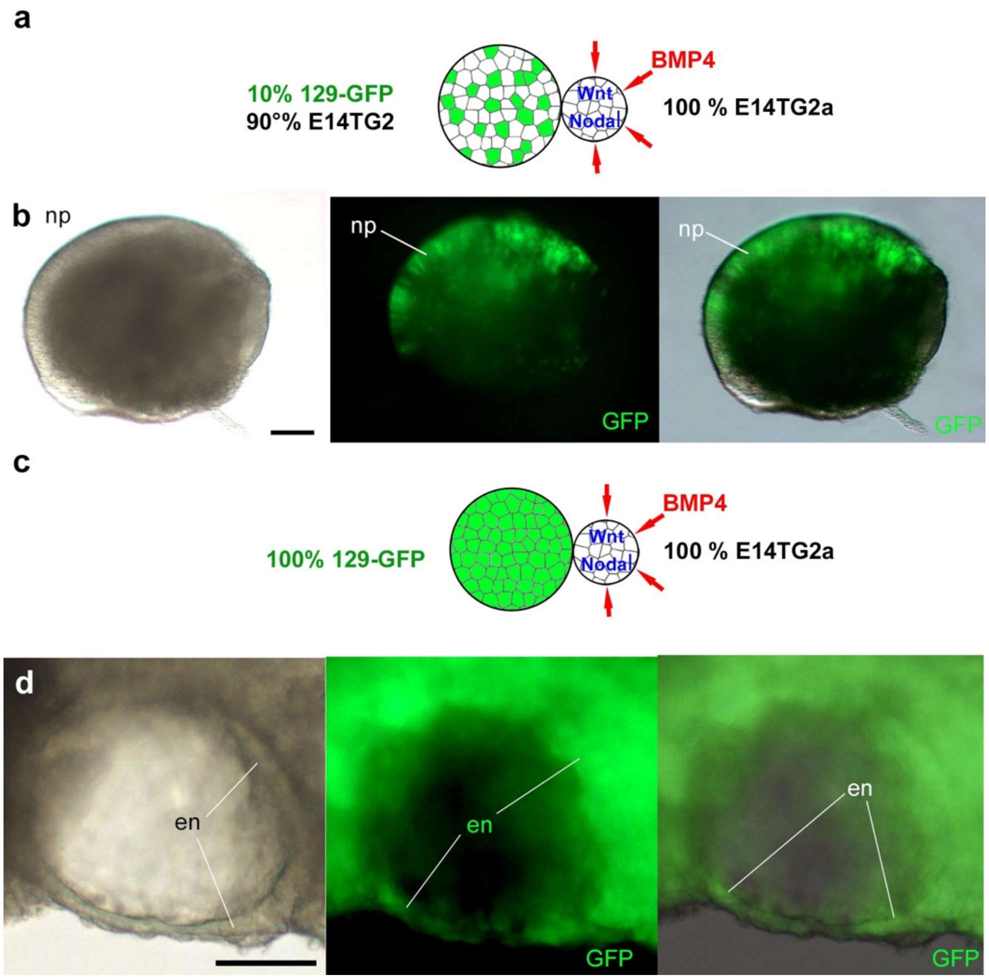

Supplementary Fig. 16: Contribution of naive cells of the embryoid to the formation of ectoderm and endoderm germ layers

(**a**) Schematic of the experiment used to generate a mosaic neural plate in an embryoid built with unlabelled E14TG2a in the signalling centre and a mixture of 90% of unlabelled cells plus 10% of 129-GFP cells in the naive portion. (**b**) Embryoid made of a naive territory with 10% 129-GFP cells/90% E14TG2 cells in brightfield (left), fluorescence (center) and a merge image of both (right) revealing that most of the fluorescent signal was present in the neural plate.

(**c**) Schematic of an experiment for which the naive domain was made with 100% 129-GFP cells and the signalling centre was made with totally unlabelled E14TG2A cells. (**d**) High magnification (brightfield - left, fluorescence - centre and a merged image (right) focusing on the endodermal epithelium (en) showing *GFP* expression in the endodermal epithelium (en). This labelling likely corresponded to VE cells deriving from GATA6(+) cells from the naive domain of the embryoids that contribute to the VE and are present in the mosaic gut epithelium made of a mix of VE and DE cells. Number of independent experiments, and the number of embryoids analysed: (**b**) Exp. = 1, N = 15/15; (**d**) Exp. = 2. N = 14/14. Scale bars: 100 µm.

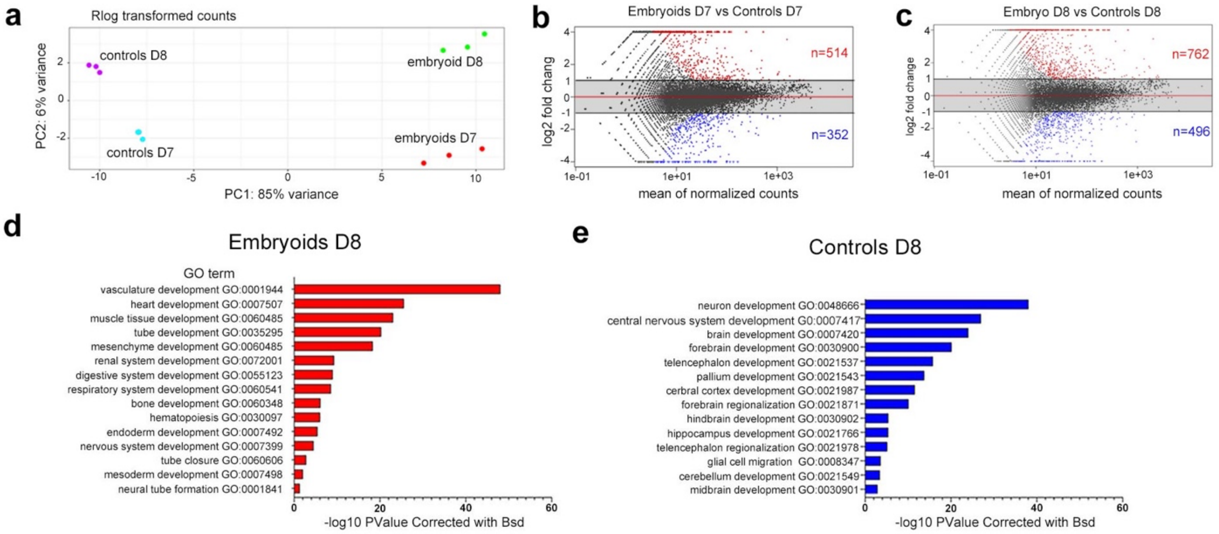

Supplementary Fig. 17: Transcriptome analysis of D7- D8 embryoids and controls

(**a**) Principal Component Analysis (PCA) of bulk RNAseq data from three replicates of 10 embryoids at D7 (red) and D8 (green) and 10 controls (made from merging at D3 of two untreated ESC aggregates) at D7 (blue) and D8 (purple). (**b**, **c**) MA plot of gene expression between embryoids and controls at (**b**) D7 and (**c**) D8. Significantly Differentially Expressed Genes (DEG) between embryoids and controls (DEG: TPM > 1, |log2FoldChange| > 1 and adjusted pvalue < 0.05); those that are upregulated are indicated in red and those that are downregulated are shown in blue. (**d, e**) Gene ontology (GO) terms enrichment analysis. For each selected biological process, the length of the bar represents (abscissa) the enrichment score [–log10(P-value corrected with Bonferroni step down)]. The red bars (**d**) represent the biological processes upregulated in embryoids at D8 while blue bars (**e**) represent the biological processes upregulated in controls at D8.

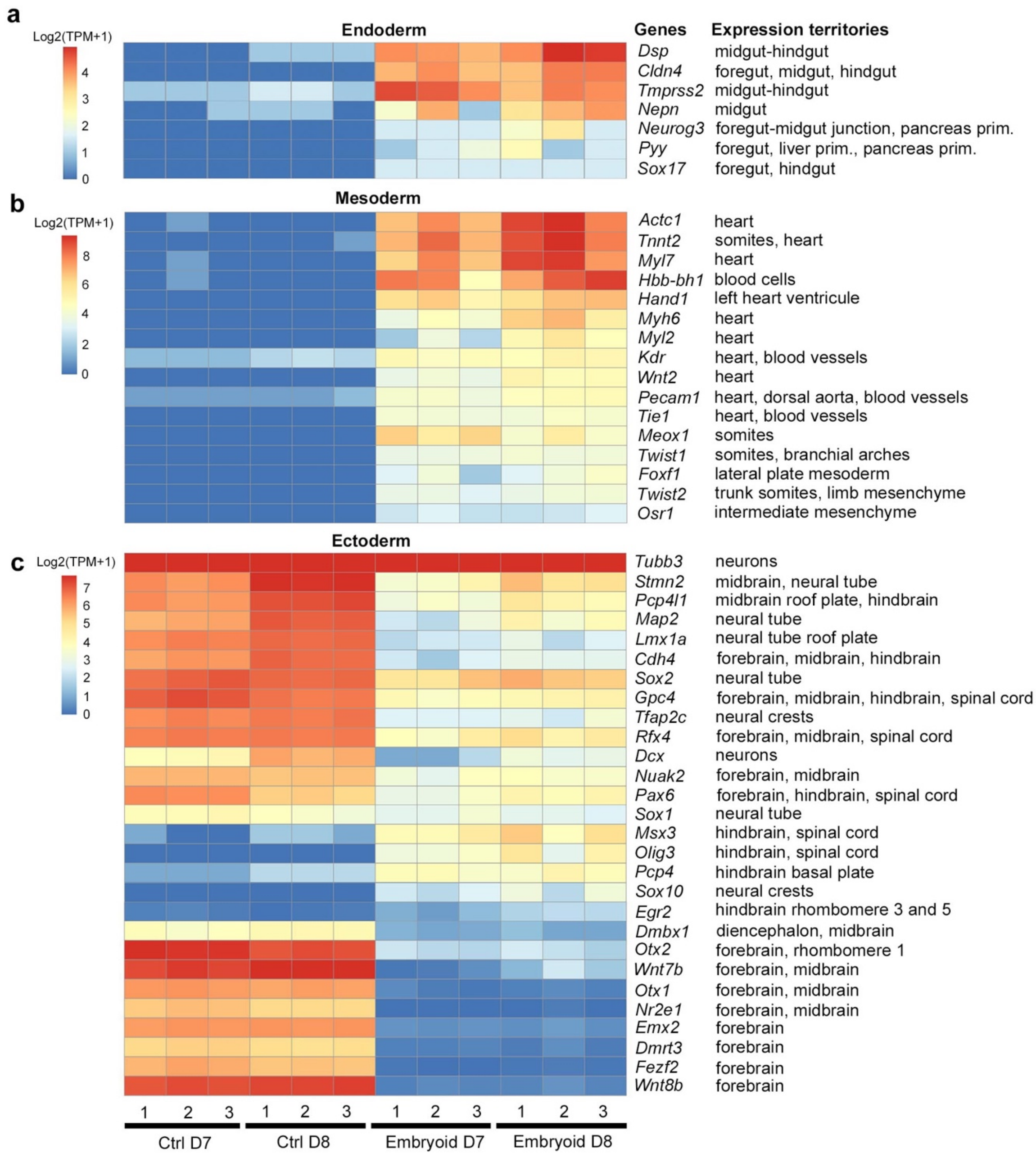

Supplementary Fig. 18: Expression profile of selected endodermal, mesodermal or ectodermal specific genes

(**a-c**) Heat maps of normalised expression at D7 and D8 in Ctrl (Controls, made of the merging at D3 of two untreated ESC aggregates) and in Embryoids, of genes that are known markers of germ layer derivatives in the mouse embryo. (**a**) Endoderm, (**b**) Mesoderm and (**c**) Ectoderm. The replicates (1, 2, 3) represented in these graphs were derived from biologically independent samples. For each gene, the expression territory (right) corresponds to the main expression domain of the gene in the mouse embryo between the embryonic stages TS13-TS15 (from 8.0 to 10.25 dpc) described in the Mouse Genome Informatics (MGI) Gene Expression Database (GXD). prim.: primordium.

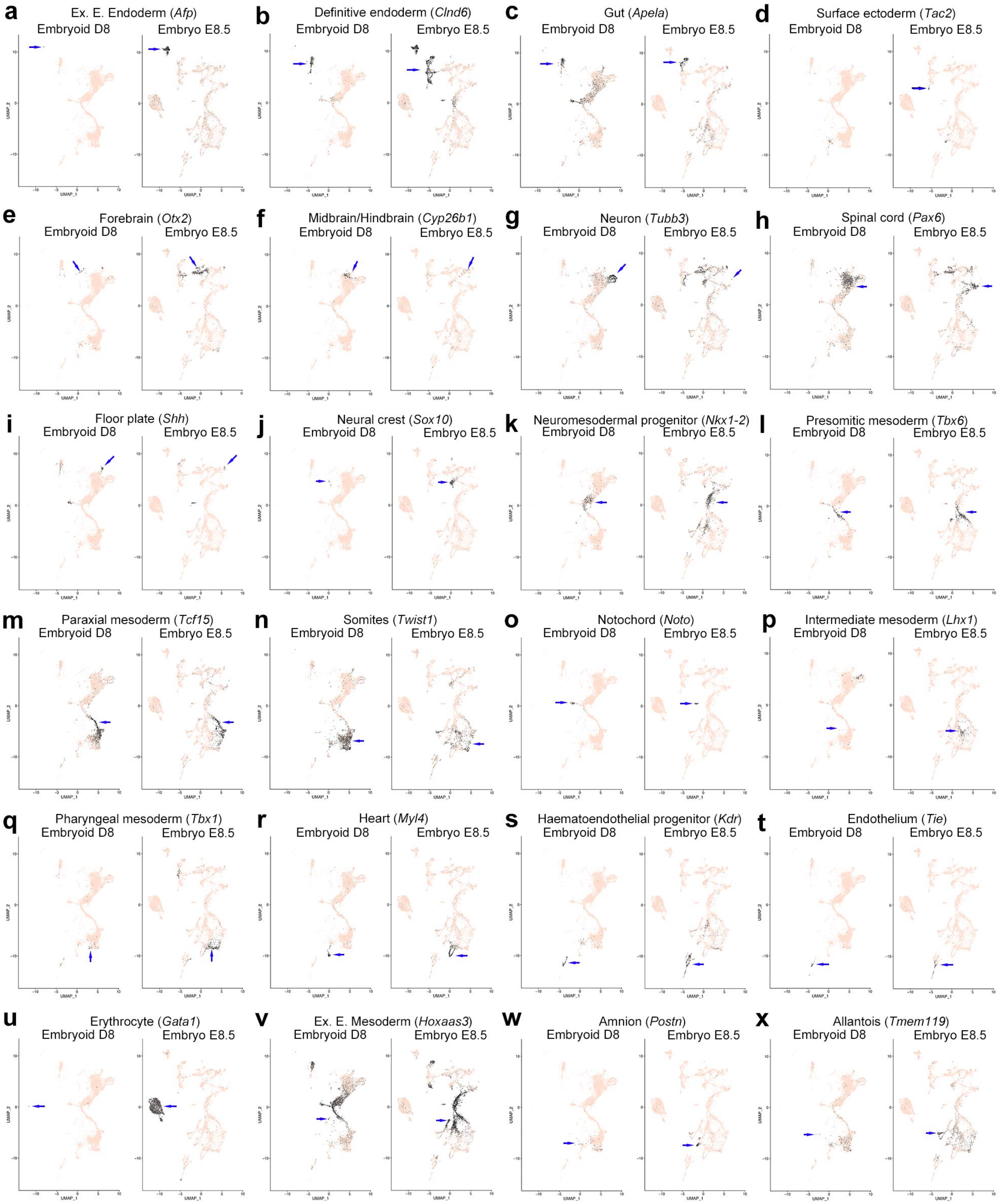

a
Ex. E. Endoderm (Afp)
b
Definitive endoderm (Clnd6)
c
Gut (Apela)
d
Surface ectoderm (Tac2)
e
Forebrain (Otx2)
f
Midbrain/Hindbrain (Cyp26b1)
g
Neuron (Tubb3)
h
Spinal cord (Pax6)
i
Floor plate (Shh)
j
Neural crest (Sox10)
k
Neuromesodermal progenitor (Nkx1-2)
l
Presomitic mesoderm (Tbx6)
m
Paraxial mesoderm (Tcf15)
n
Somites (Twist1)
o
Notochord (Noto)
p
Intermediate mesoderm (Lhx1)
q
Pharyngeal mesoderm (Tbx1)
r
Heart (Myl4)
s
Haematoendothelial progenitor (Kdr)
t
Endothelium (Tie)
u
Erythrocyte (Gata1)
v
Ex. E. Mesoderm (Hoxaas3)
w
Amnion (Postn)
x
Allantois (Tmem119)
Embryoid D8
Embryo E8.5
UMAP_1
UMAP_2

Supplementary Figure 19: UMAP plot for one marker gene of each cluster in embryoids at D8 and mouse embryos at E8.5.

(**a-x**) UMAP plot for a maker of the gene clusters (**a**) Extra-embryonic endoderm, (**b)** Definitive endoderm, (**c**) gut, (b) Surface ectoderm, (**e**) Forebrain, (**f**) Midbrain/Hindbrain, (**g**) Neuron, (**h**) Spinal cord, (**i**) Floor plate, (**j**) Neural crest, (**k**) Neuromesodermal progenitor, (**l)** Presomitic mesoderm, (**m**) paraxial mesoderm, (**n**) Somite, (**o**) Notochord, (**p**) Intermediate mesoderm, (**q**) Pharyngeal mesoderm, (**r**) Heart, (**s**) Haematoendothelial progenitors, (**t**) Endothelium, (**u**) Erythrocyte, (**v**) Extra-embryonic mesoderm, (**w**) Amnion, (**x**) Allantois. The names of the cluster and of the marker gene identifying this cluster are indicated at the top of each panel. The position of identified cells of each cluster is indicated with a blue arrow for both embryoids and embryos.

a

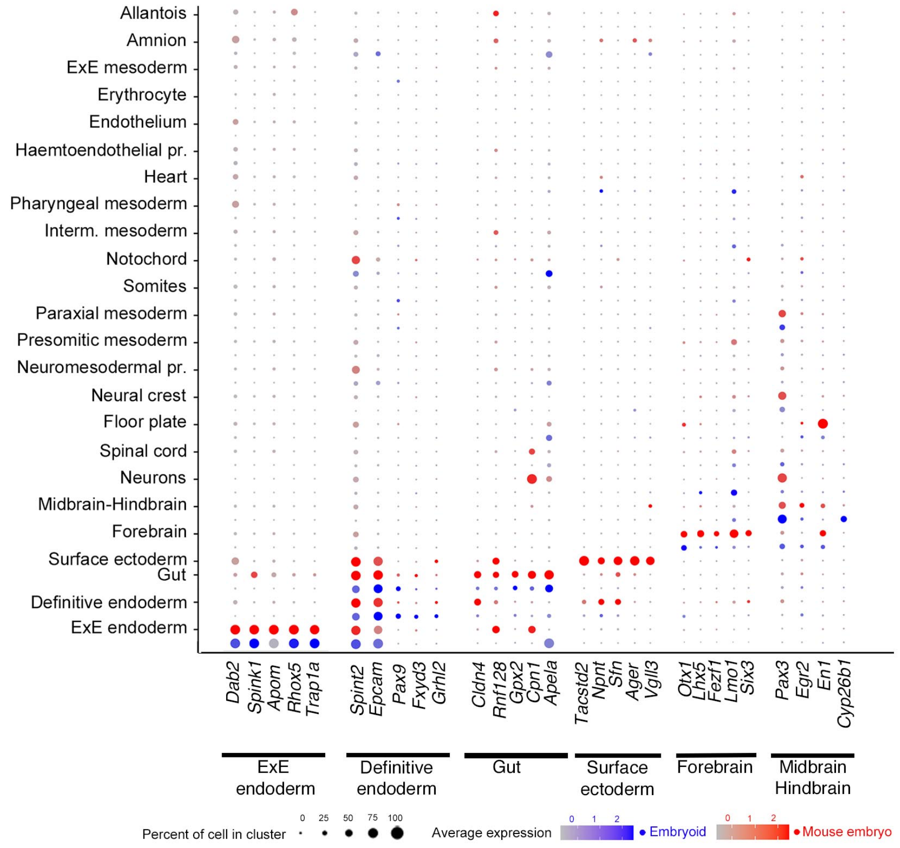
Allantois
Amnion
ExE mesoderm
Erythrocyte
Endothelium
Haemtoendothelial pr.
Heart
Pharyngeal mesoderm
Interm. mesoderm
Notochord
Somites
Paraxial mesoderm
Presomitic mesoderm
Neuromesodermal pr.
Neural crest
Floor plate
Spinal cord
Neurons
Midbrain-Hindbrain
Forebrain
Surface ectoderm
Gut
Definitive endoderm
ExE endoderm
Dab2
Spink1
Apom
Rhox5
Trap1a
Spint2
Epcam
Pax9
Fxyd3
Grhl2
Cldn4
Rnf128
Gpx2
Cpn1
Apela
Tacstd2
Npnt
Sfn
Ager
Vgll3
Otx1
Lhx5
Fezf1
Lmo1
Six3
Pax3
Egr2
En1
Cyp26b1
ExE endoderm
Definitive endoderm
Gut
Surface ectoderm
Forebrain
Midbrain Hindbrain
Percent of cell in cluster
0 25 50 75 100
Average expression
0 1 2
Embryoid
0 1 2
Mouse embryo

b

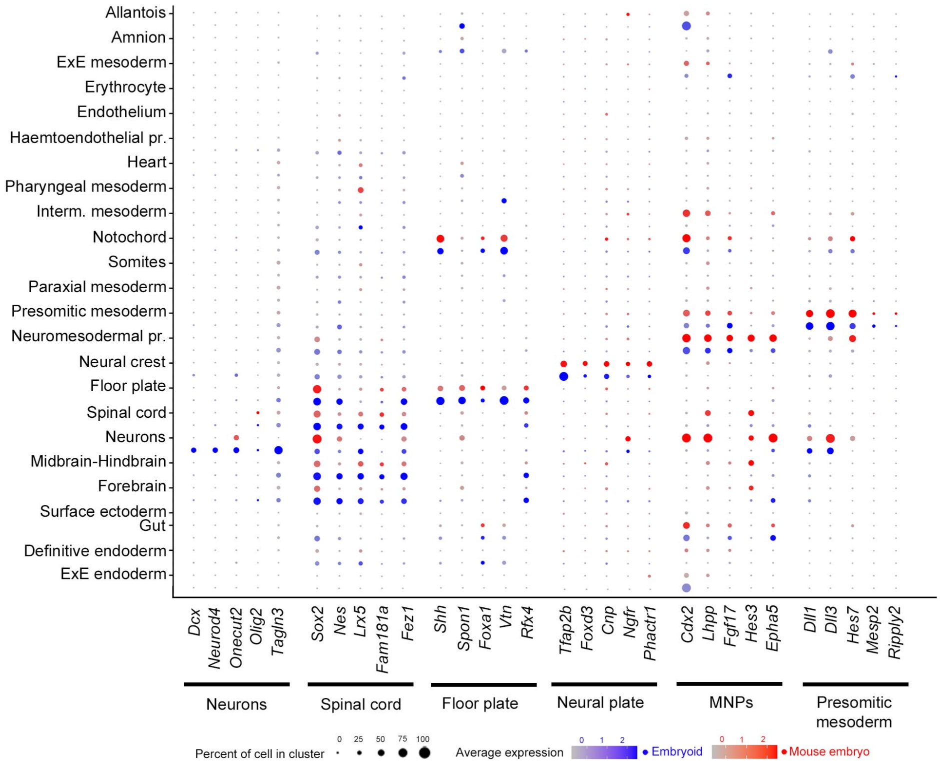
Allantois
Amnion
ExE mesoderm
Erythrocyte
Endothelium
Haemtoendothelial pr.
Heart
Pharyngeal mesoderm
Interm. mesoderm
Notochord
Somites
Paraxial mesoderm
Presomitic mesoderm
Neuromesodermal pr.
Neural crest
Floor plate
Spinal cord
Neurons
Midbrain-Hindbrain
Forebrain
Surface ectoderm
Gut
Definitive endoderm
ExE endoderm
Dcx
Neurod4
Onecut2
Olig2
Tagln3
Sox2
Nes
Lrx5
Fam181a
Fez1
Shh
Spon1
Foxa1
Vtn
Rfx4
Tfap2b
Foxd3
Cnp
Ngfr
Phactr1
Cdx2
Lhpp
Fgf17
Hes3
Epha5
Dll1
Dll3
Hes7
Mesp2
Ripply2
Neurons
Spinal cord
Floor plate
Neural plate
MNPs
Presomitic mesoderm
Percent of cell in cluster
0 25 50 75 100
Average expression
0 1 2
Embryoid
0 1 2
Mouse embryo

Supplementary Fig. 20: Dot plot graph comparing expression of specific marker gene between embryos and embryoids

Dot plot showing both the percentage of cells (diameter of the dot) of a given cluster that expressed the marker gene of that cluster and its level of expression (gradient of colour, blue for embryoids, red for mouse embryos).

(**a**) Marker genes for clusters Exe Endoderm, Definitive endoderm, Gut, Surface ectoderm, Forebrain, Midbrain-Hindbrain.

(**b**) Marker genes for clusters Neurons, Spinal cord, Floor plate, Neural plate, Neuromesodermal progenitors and Presomitic mesoderm.

Name of clusters is shown at the left and the name of marker genes for each cluster is shown at the bottom. For each cluster, mouse embryo data are shown in red and embryoid data are shown in blue. Exe: extra embryonic.

a

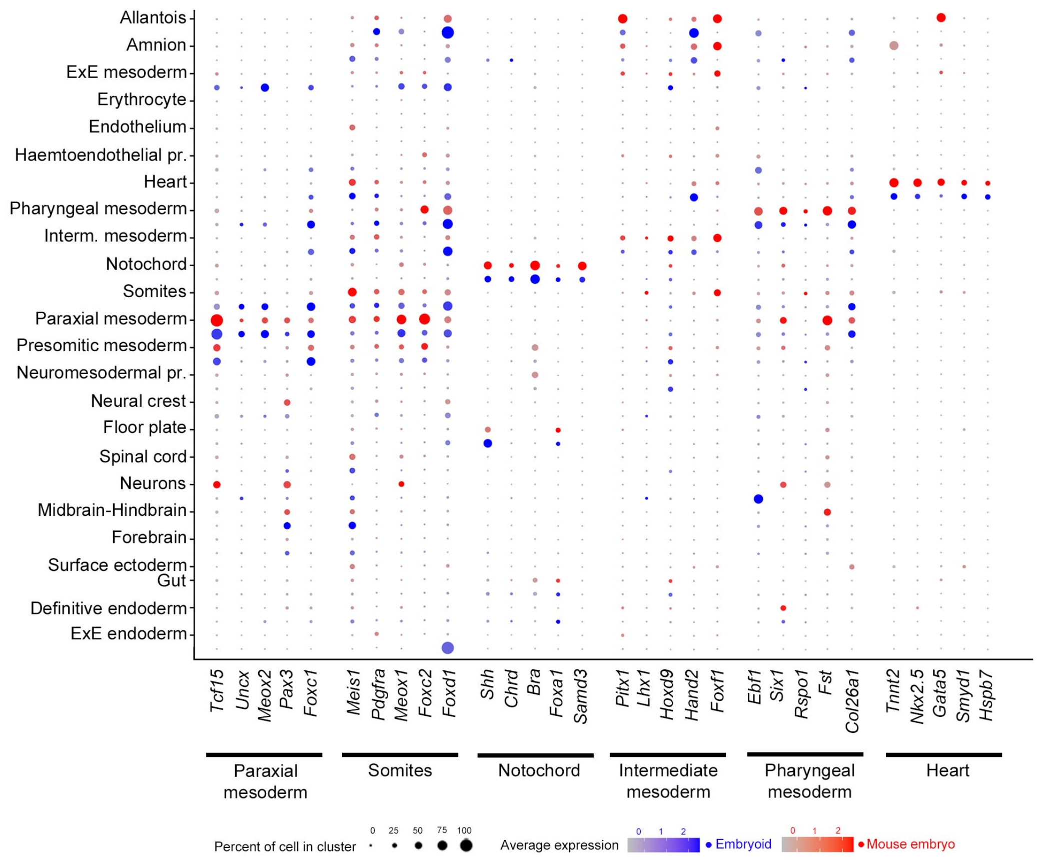
Allantois
Amnion
ExE mesoderm
Erythrocyte
Endothelium
Haemtoendothelial pr.
Heart
Pharyngeal mesoderm
Interm. mesoderm
Notochord
Somites
Paraxial mesoderm
Presomitic mesoderm
Neuromesodermal pr.
Neural crest
Floor plate
Spinal cord
Neurons
Midbrain-Hindbrain
Forebrain
Surface ectoderm
Gut
Definitive endoderm
ExE endoderm
Tcf15
Uncx
Meox2
Pax3
Foxc1
Meis1
Pdgfra
Meox1
Foxc2
Foxd1
Shh
Chrd
Bra
Foxa1
Samd3
Pitx1
Lhx1
Hoxd9
Hand2
Foxf1
Ebf1
Six1
Rspo1
Fst
Col26a1
Tnnt2
Nkx2.5
Gata5
Smyd1
Hspb7
Paraxial mesoderm
Somites
Notochord
Intermediate mesoderm
Pharyngeal mesoderm
Heart
Percent of cell in cluster
0
25
50
75
100
Average expression
0
1
2
Embryoid
0
1
2
Mouse embryo

b

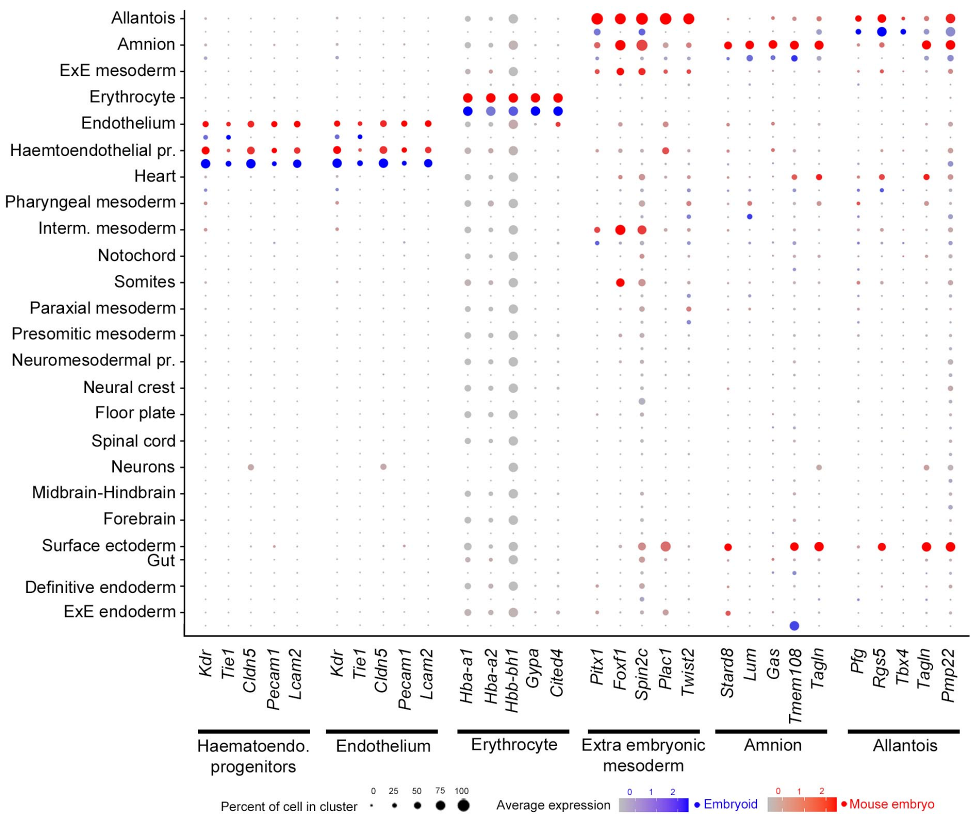
Allantois
Amnion
ExE mesoderm
Erythrocyte
Endothelium
Haemtoendothelial pr.
Heart
Pharyngeal mesoderm
Interm. mesoderm
Notochord
Somites
Paraxial mesoderm
Presomitic mesoderm
Neuromesodermal pr.
Neural crest
Floor plate
Spinal cord
Neurons
Midbrain-Hindbrain
Forebrain
Surface ectoderm
Gut
Definitive endoderm
ExE endoderm
Kdr
Tie1
Cldn5
Pecam1
Lcam2
Kdr
Tie1
Cldn5
Pecam1
Lcam2
Hba-a1
Hba-a2
Hbb-bh1
Gypa
Cited4
Pitx1
Foxf1
Spin2c
Plac1
Twist2
Stard8
Lum
Gas
Tmem108
Tagln
Pfg
Rgs5
Tbx4
Tagln
Pmp22
Haematoendo. progenitors
Endothelium
Erythrocyte
Extra embryonic mesoderm
Amnion
Allantois
Percent of cell in cluster
0
25
50
75
100
Average expression
0
1
2
Embryoid
0
1
2
Mouse embryo

Supplementary Fig. 21: Dot plot graph comparing expression of specific marker gene between embryos and embryoids

Dot plot showing both the percentage of cells (diameter of the dot) of a given cluster that expressed the marker gene of that cluster and its level of expression (gradient of colour, blue for embryoids, red for mouse embryos).

(**a**) Marker genes for clusters Paraxial mesoderm, Somites, Notochord, Intermediate mesoderm, Pharyngeal mesoderm and Heart.

(**b**) Marker genes for clusters Haematoendothelial progenitors, Endothelium, Erythrocyte, Extra embryonic mesoderm, Amnion and Allantois.

Name of clusters is shown at the left and the name of marker genes for each cluster is shown at the bottom. For each cluster, mouse embryo data are shown in red and embryoid data are shown in blue. Exe: extra embryonic.

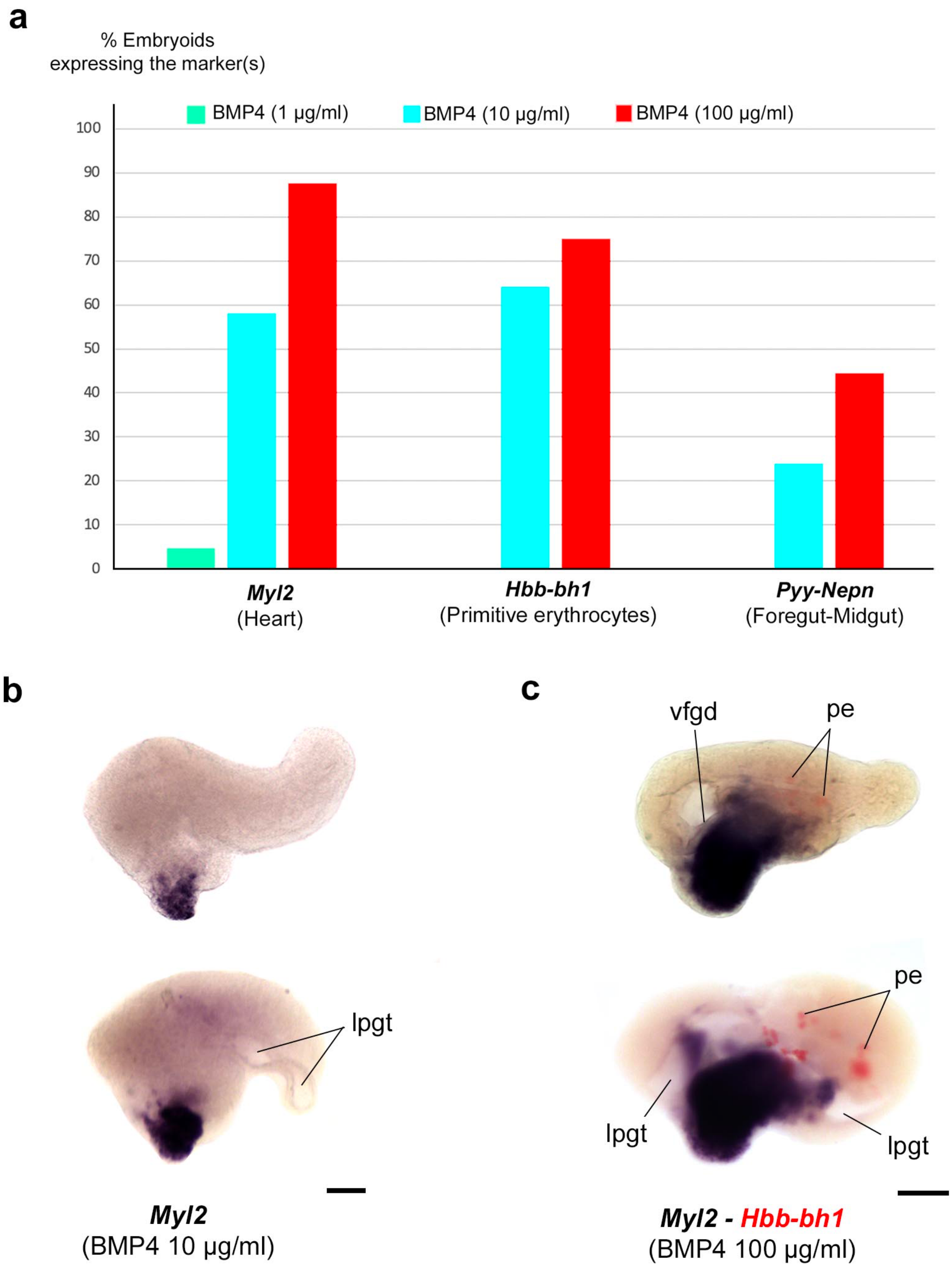

Supplementary Fig. 22: Effect of BMP4 concentration on the frequency of expression of molecular markers and on the size of the heart and the gut tube

(**a**) Graph bars representing the percentage of embryoids expressing the heart marker *Myl2*, the primitive erythrocyte (pe) marker *Hbb-bh1* and the gut epithelium markers *Pyy* and *Nepn* as a function of the BMP4 concentration used for the instruction of the signalling centre at D2.66-D3.

Number of embryoids analysed in two experiments for the expression of *Myl2*, n=21 for 1µg/ml BMP4, N=106 for 10µg/ml BMP4 and N=16 for 100 µg/ml BMP4. For the expression of *Hbb-bh1*: N=/42 for 1µg/ml BMP4, N=181 for 10µg/ml BMP4 and N=16 for 100 µg/ml BMP4. For the expression of *Pyy* and *Nepn:* N=19 for 1µg/ml BMP4, N=42 for 10µg/ml BMP4 and N=27 for 100 µg/ml BMP4. (**b**) Single colour *in situ* hybridization for *Myl2* in D8 embryoids built with a signalling centre instructed by incubation with 10µg/ml BMP4. The gut epithelium is clearly visible (lpgt: lumen of the primitive gut tube). (**c**) Double colour *in situ* hybridization for *Myl2* (blue) and *Hbb-bh1* (red, pe: primitive erythrocytes) in D8 embryoids built with a signalling centre instructed by incubation with 100µg/ml BMP4. The heart as well as the lumen of the primitive gut tube are strongly enlarged compared to (**b**). vfgd: ventral foregut diverticulum. Scale bars: 100µm

Supplementary Table 1: Efficacy of merging of ESC aggregates

Experiments	Merged aggregates	Total attempts of merging	% merged
1	187	192	97.40
2	576	576	100.00
3	382	384	99.48
4	576	576	100.00
5	381	384	99.22
6	574	576	99.65
7	573	576	99.48
8	478	480	99.58
9	286	288	99.31
10	567	576	98.44
11	574	576	99.65
Total	5154	5184	
Mean			99.29
Standard Deviation			1.59

Supplementary Table 2: Marker genes used to identify the different cell clusters

Cluster Identity		Cluster	marker genes	
Allantois	*Twist2*	*Tmem119*	*Plac1*	
Amnion	*Tdo2*	*Postn*	*Lum*	
Extra embryonic mesoderm	*Hand1*	*Hoxaas3*		
Erythrocyte	*Gata1*	*Hbb-bh1*	*Gypa*	*Klf1*
Endothelium	*Pecam1*	*Tie1*		
Haematoendothelial progenitors	*Etv2*	*Kdr*		
Heart	*Gata4*	*Tbx20*	*Myl2*	*Nkx2-5*
Pharyngeal mesoderm	*Fst*	*Eb2*	*Tbx1*	
Intermediate mesoderm	*Osr1*	*Lhx1*		
Notochord	*Noto*	*Chrd*	*Bra/T*	*Shh*
Somites	*Foxd1*	*Twist1*	*Pax9*	
Paraxial mesoderm	*Pax1*	*Uncx*	*Tcf15*	*Sox21*
Presomitic mesoderm	*Tbx6*	*Mesp1*	*Ripply2*	*Lnfg*
Neuromesodermal progenitors	*Nkx1-2*	*Wnt3a*	*Cdx2*	
Neural crest	*Sox10*	*Foxd3*	*Ets1*	*Tfab2b*
Floor plate	*Slit1*	*Spon1*	*Shh*	*Foxa2*
Spinal cord	*Sox2*	*Pax6*	*Pax3*	*Nes*
Neurons	*Tubb3*	*Dcx*	*Onecut2*	
Midbrain/Hindbrain	*Lrx2*	*Cyp26b1*	*Sox21*	
Forebrain	*Otx2*	*Six3*	*Lhx2*	
Surface ectoderm	*Tac2*	*Tacstd2*	*Ager*	
Gut	*Sox17*	*Apela*	*Rnf128*	*Nepn*
Definitive endoderm	*Cldn6*	*Six1*	*Tbx1*	*Wnt6*
Extra embryonic endoderm	*Apoa1*	*Afp*	*Dab2*	

Supplementary Table 3: Number of cells present in each cluster for mouse embryos at E8.5 and embryoids at D8

	Embryo E8.5	Embryo E8.5	Embryoid D8	Embryoid D8
Cluster name	Number of cells	Percentage	Number of cells	Percentage
Forebrain	647	7.53	24	0.36
Midbrain-Hindbrain	491	5.72	278	4.19
Spinal cord	259	3.02	1890	28.47
Floor plate	48	0.56	72	1.08
Neural crest	322	3.75	8	0.12
Neurons	2	0.02	357	5.38
Neuromesodermal progenitors	556	6.47	1028	15.48
Notochord	38	0.44	125	1.88
Presomitic mesoderm	295	3.43	302	4.55
Paraxial mesoderm	241	2.81	545	8.21
Somite	242	2.82	1125	16.95
Intermediate mesoderm	609	7.09	15	0.23
Pharyngeal mesoderm	158	1.84	266	4.01
Heart	392	4.56	187	2.82
Haematoendothelial progenitors	287	3.34	69	1.04
Endothelium	81	0.94	3	0.05
Definitive endoderm	531	6.18	34	0.51
Gut	205	2.39	290	4.37
Extra embryonic endoderm	327	3.81	1	0.02
Extra embryonic mesoderm	373	4.34	12	0.18
Amnion	235	2.74	5	0.08
Allantois	188	2.19	2	0.03
Erythrocyte	2017	23.48	1	0.02
Surface Ectoderm	46	0.54	0	-
Total	8590	100.00	6639	100.00

Supplementary Table 4: Nucleotide sequences of oligonucleotides used for the RT-PCR experiment (Supplementary Figure 1)

Gene	Oligonucleotide sequence
Bra/T F	CTCGGATTCACATCGTGAGAG
Bra/T R	AAGGCTTTAGCAAATGGGTTGTA
Eomes F	CCTGGTGGTGTTTTGTTGTG
Eomes R	TTTAATAGCACCGGGCACTC
Gata6 F	TTGCTCCGGTAACAGCAGTG
Gata6 R	GTGGTCGCTTGTGTAGAAGGA
Kdr F	GGGTCGATTTCAAACCTCAATGT
Kdr R	AGAGTAAAGCCTATCTCGCTGT
Nanog F	TTGCTTACAAGGGTCTGCTACT
Nanog R	ACTGGTAGAAGAATCAGGGCT
Nodal F	CCTGGAGCGCATTTGGATG
Nodal R	ACTTTTCTGCTCGACTGGACA
Pou5f1 F	AGTTGGCGTGGAGACTTTGC
Pou5f1 R	CAGGGCTTTCATGTCCTGG
Sox17 F	CGAGCCAAAGCGGAGTCTC
Sox17 R	TGCCAAGGTCAACGCCTTC
Wnt3 F	ACGCCCCTTTCTGTTGTTCT
Wnt3 R	GGGTAGTCTGCCTTCTAACACA

F: forward primer, R: reverse primer

Supplementary table 5: Expression territories in the mouse embryo of the molecular markers used in this study

Genes *In situ*	Other labelling	Expression territories	references
Apela		Ventral foregut diverticulum, hindgut diverticulum	1
Bra/T		primitive streak, notochord	2
	Bra-GFP	primitive streak, mesoderm cells at gastrulation, notochord	3
Cdx2		posterior spinal cord	4
Cer1		anterior compartment of the newly formed somite and in the anterior adjacent unsegmented PSM	5
Chrd		Notochord, node	6
Cldn4		Gut endoderm	7
	DBA lectin	visceral endoderm	8
Egr2		Rhombomere 3 and 5 of the rhombencephalon	9
En2		Midbrain, midbrain-hindbrain boundary	10
eomes		Mesoderm gastrulation	11
Fgf8		forebrain, isthmus,	12
FoxJ1		Node	13
Gata6		Primitive and Visceral endoderm	14
Hbb-bh1		Primitive erythrocytes	15
Hoxd4		anterior limit of the rhombomere 6/7	16
Hoxd9		tail bud	17
Kdr		Vessel-derived endothelial cells	18
Meox1		Paraxial mesoderm	19
Mesp1		Mesoderm gastrulation, anterior PSM	11
Myl2		Heart primitive ventricule	20
NANOG	NANOG (Ab)	Epiblast	14
Nepn		Midgut epithelium	21
Nkx1-2		caudal lateral epiblast	22
Nodal		Primitive streak, node	23
Noto		Posterior notochord	24
Olig2		Motor neurons and oligodendrocytes	25
Osr1		Intermediate mesoderm	26
Otx2		Anterior neurectoderm after TS10	27
Pax6		Forebrain, eye, hindbrain and spinal chord	28
Pyy		Foregut pocket epithelium	21
Runx1		proximal yolk sac mesoderm	29
Scube2		forebrain, hindbrain, spinal cord	30
Shh		Notochord, floor plate	31
Smarcd3		cardiac crescent	32
	Sox1-GFP	Neurectoderm	33
Sox10		Neural crest	34
Sox17		Endoderm	35
Sox2		Neurectoderm	36
Wnt3		primitive streak	37
Wnt3a		Posterior primitive streak	38

Supplementary references

1 Hassan, A.S. et al. Expression of two novel transcripts in the mouse definitive endoderm. *Gene Expr. Patterns* **10**, 127-34 (2010).

2 Andersson, O. et al. Synergistic interaction between Gdf1 and Nodal during anterior axis development. *Dev. Biol.* **293**, 370-81 (2006).

3 Fehling, H. J*, et al.* Tracking mesoderm induction and its specification to the hemangioblast during embryonic stem cell differentiation. *Development* **130**, 4217-4227 (2003).

4 Savory, J. G. et al. Cdx2 regulation of posterior development through non-Hox targets *Development* **136**, 4099-110 (2009).

5 Belo J. A. et al. Cerberus-like is a secreted factor with neutralizing activity expressed in the anterior primitive endoderm of the mouse gastrula. *Mech. Dev.* **68**, 45-57 (1997).

6 Rhinn, M. et al. Sequential roles for Otx2 in visceral endoderm and neuroectoderm for forebrain and midbrain induction and specification. *Development*. **125**,-56 (1998).

7 Tamplin, O. J. et al. Microarray analysis of Foxa2 mutant mouse embryos reveals novel gene expression and inductive roles for the gastrula organizer and its derivatives. *BMC Genomics* **9**, 511 (2008).

8 Polydorou, C. & Georgiades, P. Ets2-dependent trophoblast signalling is required for gastrulation progression after primitive streak initiation. *Nat. Commun* **4**, 1658 (2013).

9 Mechta-Grigoriou, F. et al. Nab proteins mediate a negative feedback loop controlling Krox-20 activity in the developing hindbrain. *Development* 127,119-28 (2000).

10 Li Song, D. et al., Two Pax2/5/8-binding sites in Engrailed2 are required for proper initiation of endogenous mid-hindbrain expression. *Mech. Dev*. **90**, 155-65 (2000).

11 Costello, I., Pimeisl, I. M., Dräger, S., Bikoff, E. K., Robertson, E. J. & Arnold, S. J. The T-box transcription factor Eomesodermin acts upstream of Mesp1 to specify cardiac mesoderm during mouse gastrulation *Nat Cell Biol* **1**, 1084-91 (2011).

12 Chi, C. L., et al. The isthmic organizer signal FGF8 is required for cell survival in the prospective midbrain and cerebellum. *Development*. **130**, 2633-44 (2003).

13 Cruz, C. et al. Foxj1 regulates floor plate cilia architecture and modifies the response of cells to sonic hedgehog signalling. *Development*. **137**, 4271-82 (2010).

14 Schrode, N., Saiz, N., Di Talia, S. & Hadjantonakis, A. K. GATA6 levels modulate primitive endoderm cell fate choice and timing in the mouse blastocyst. *Dev. Cell*. **29**, 454-67 (2014).

15 Silver, L. & Palis J. Initiation of murine embryonic erythropoiesis: a spatial analysis. *Blood* **89**, 1154-64 (1997).

16 Bel-Vialar, S. et al. Altered retinoic acid sensitivity and temporal expression of Hox genes in polycomb-M33-deficient mice. *Dev. Biol*. **224**, 238-4 (2000).

17 Nowotschin, S. et al. The emergent landscape of the mouse gut endoderm at single-cell resolution. *Nature* **569**, 361-367 (2019).

18 Yamaguchi T. P. et al. flk-1, an flt-related receptor tyrosine kinase is an early marker for endothelial cell precursors. *Development*. **118**, 489-98 (1993).

19 Conlon, R.A. et al. Notch1 is required for the coordinate segmentation of somites. *Development*. **121**, 1533-45 (1995).

20 Vong, L. et al. MEF2C is required for the normal allocation of cells between the ventricular and sinoatrial precursors of the primary heart field. *De.v Dyn.* **235**, 1809-21 (2006).

21 Hou, J. et al. A systematic screen for genes expressed in definitive endoderm by Serial Analysis of Gene Expression (SAGE). *BMC Dev. Bio.l* **7**, 92 (2007).

22 Rodrigo Albors, A., Halley, P. A., Storey, K. G. Development. Lineage tracing of axial progenitors using Nkx1-2CreER T2 mice defines their trunk and tail contributions. *Development* **145**, dev164319 (2018).

23 Lu, C. C. et al., Multiple roles for Nodal in the epiblast of the mouse embryo in the establishment of anterior-posterior patterning. *Dev. Biol.* **273**, 149-59 (2004).

24 Abdelkhalek, H. B et al. The mouse homeobox gene Not is required for caudal notochord development and affected by the truncate mutation. *Genes Dev* **18**, 1725-36 (2004).

25 Molotkova, N. et al. Requirement of mesodermal retinoic acid generated by Raldh2 for posterior neural transformation. *Mech. Dev*. **122**, 145-55 (2005).

26 So, P. L. et al. Cloning and expression analysis of a mouse gene related to Drosophila odd-skipped. *Mech. Dev.* **84**, 157-60 (1999).

27 Ang, S. L. et al. Positive and negative signals from mesoderm regulate the expression of mouse Otx2 in ectoderm explants. *Development*. **120**, 2979-89 (1994).

28 Anderson, T. R. et al. Differential Pax6 promoter activity and transcript expression during forebrain development. *Mech. Dev.* **114**, 171-5 (2002).

29 Lacaud, G. et al. Runx1 is essential for hematopoietic commitment at the hemangioblast stage of development in vitro. *Blood* **100**, 458-66 (2002).

30 Grimmond, S. et al. Expression of a novel mammalian epidermal growth factor-related gene during mouse neural development *Mech. Dev*. **102**, 209-11 (2001).

31 Echelard, Y. et al. Sonic hedgehog, a member of a family of putative signaling molecules, is implicated in the regulation of CNS polarity. *Cell*. **75**, 1417-30 (1993).

32 Zhao, R. et al. Loss of both GATA4 and GATA6 blocks cardiac myocyte differentiation and results in acardia in mice. *Dev. Biol.* **317**, 614–619 (2008).

33 Aubert, J. et al. *Proc. Nat.l Acad. Sc.i U S A*. **100 Suppl**, 111836-41 (2003).

34 Anderson, R. M. et al. Endogenous bone morphogenetic protein antagonists regulate mammalian neural crest generation and survival. *Dev. Dyn*. **235**, 2507-2520 (2006).

35 Artus, J., Piliszek, A. & Hadjantonakis, A. K. The primitive endoderm lineage of the mouse blastocyst: sequential transcription factor activation and regulation of differentiation by Sox17. *Dev. Biol*. **350**, 393-404 (2011).

36 Wood, H. B. et al. Comparative expression of the mouse Sox1, Sox2 and Sox3 genes from pre-gastrulation to early somite stages. *Mech. Dev.* **86**, 197-201 (1999).

37 Rivera-Pérez, J. A. & Magnuson, T. Primitive streak formation in mice is preceded by localized activation of Brachyury and Wnt3. *Dev. Biol*. **288**, 363-71 (2005).

38 Parr, B. A. et al. Mouse Wnt genes exhibit discrete domains of expression in the early embryonic CNS and limb buds. *Development*. **119**, 247-261 (1993).
