# Supplementary material for: Construction of a mammalian embryo model from stem cells organized by a morphogen signalling centre
Source: Nat Commun. 2021 Jun 2;12:3277. doi: 10.1038/s41467-021-23653-4 (PMC8172561; doi:10.1038/s41467-021-23653-4)
Supplement: Supplementary file 2 — Reporting Summary [file 41467_2021_23653_MOESM2_ESM.pdf]

nature research

Corresponding author(s): Christine Thisse

Last updated by author(s): Apr 27, 2021

Reporting Summary

Nature Research wishes to improve the reproducibility of the work that we publish. This form provides structure for consistency and transparency in reporting. For further information on Nature Research policies, see our Editorial Policies and the Editorial Policy Checklist.

Statistics

For all statistical analyses, confirm that the following items are present in the figure legend, table legend, main text, or Methods section.

n/a	Confirmed	
☐	☒	The exact sample size (*n*) for each experimental group/condition, given as a discrete number and unit of measurement
☐	☒	A statement on whether measurements were taken from distinct samples or whether the same sample was measured repeatedly
☐	☒	The statistical test(s) used AND whether they are one- or two-sided<br>*Only common tests should be described solely by name; describe more complex techniques in the Methods section.*
☒	☐	A description of all covariates tested
☒	☐	A description of any assumptions or corrections, such as tests of normality and adjustment for multiple comparisons
☐	☒	A full description of the statistical parameters including central tendency (e.g. means) or other basic estimates (e.g. regression coefficient) AND variation (e.g. standard deviation) or associated estimates of uncertainty (e.g. confidence intervals)
☐	☒	For null hypothesis testing, the test statistic (e.g. *F*, *t*, *r*) with confidence intervals, effect sizes, degrees of freedom and *P* value noted<br>*Give P values as exact values whenever suitable.*
☒	☐	For Bayesian analysis, information on the choice of priors and Markov chain Monte Carlo settings
☒	☐	For hierarchical and complex designs, identification of the appropriate level for tests and full reporting of outcomes
☒	☐	Estimates of effect sizes (e.g. Cohen's *d*, Pearson's *r*), indicating how they were calculated

Our web collection on statistics for biologists contains articles on many of the points above.

Software and code

Policy information about availability of computer code

Data collection	CoolSNAP version 1.2; leica application suite Advance Fluorescence 2.7.2.9586
Data analysis	image J 1.48v; R version 3.5.1.; Microsoft Excel 2016, Leica Application suite X (LAS X), Prism 9 v9.0.0.; Photoshop cc2019; For Bulk RNAseq analysis Sequencing reads were filtered and mapped to the mouse genome build mm10 using the STAR alignment program (v2.6.1a). Principal Component Analysis and Differentially Expressed Genes were calculated using the R package DESeq2 845 (v1.22.2). Heatmaps were generated using the R package pheatmap (v1.0.12). For single –cell RNAseq experiments data set was processed with Cell Ranger 3.1.0. Quality control and cluster determination was performed using the Seurat package (V3.2.1) in Rstudio (V1.3.1093). Pseudotime analysis was performed using the R Package Monocle 3.

For manuscripts utilizing custom algorithms or software that are central to the research but not yet described in published literature, software must be made available to editors and reviewers. We strongly encourage code deposition in a community repository (e.g. GitHub). See the Nature Research guidelines for submitting code & software for further information.

Data

Policy information about availability of data

All manuscripts must include a data availability statement. This statement should provide the following information, where applicable:

- Accession codes, unique identifiers, or web links for publicly available datasets
- A list of figures that have associated raw data
- A description of any restrictions on data availability

The source data underlying Figures are provided as Supplementary Figures describing the number of experiments, the number in biological samples analysed and a

description of variability of gene expression pattern. All other data are available in the main text (Fig.1-10) or the Supplementary Figures (1 - 21), Supplementary Tables (1 - 7) and Raw data are provided in Source Data File for Supplementary Figures 1, 2, 3, 4, 6 and 13 . Bulk and scRNAseq data have been deposited at the National Center for Biotechnology Information Gene Expression Omnibus under accession number: GSE142309 for the Bulk RNA sequencing, https://www.ncbi.nlm.nih.gov/geo/query/acc.cgi?acc=GSE142309 and GSE168539 for the single cell RNA sequencing,

Field-specific reporting

Please select the one below that is the best fit for your research. If you are not sure, read the appropriate sections before making your selection.

[x] Life sciences [ ] Behavioural & social sciences [ ] Ecological, evolutionary & environmental sciences

For a reference copy of the document with all sections, see nature.com/documents/nr-reporting-summary-flat.pdf

Life sciences study design

All studies must disclose on these points even when the disclosure is negative.

Sample size	For in situ hybridization and immunolabelling the number of embryoids analyzed has been defined such that we can detect at least variation in 10% of individuals - more than 15 for in situ hybridization, at least 5 for immunolabelling analysed by confocal imaging. For bulk RNAseq the number of embryoids (batches of 10) has been choosen based on the needed amount of isolated RNA. For single-cell RNA seq, the number of embryoids (90) has been choosen to allow recovery of at least 10,000 cells for analysis.
Data exclusions	For single cell RNA sequencing, cells with low library complexity (under 1,700 genes and mitochondrial-fraction greater than 5%) were excluded (cf reference: Satija, R., Farrell, J. A., Gennert, D., Schier, A.F . & Regev, A. Spatial reconstruction of single-cell gene expression data. Nat. Biotechnol. 33; 495-502 (2015).)
Replication	labelling on embryoids has been performed multiple times for each probe as reported in Statistics and Reproducibility in the method section. All attempts at replication were successful. Bulk RNA seq has been performed on three replicates with highly similar expression results in the three transcriptomes of each stages analyzed (D7, D8)
Randomization	Not relevant because there were no experimental groups
Blinding	Not relevant because there were no experimental groups

Reporting for specific materials, systems and methods

We require information from authors about some types of materials, experimental systems and methods used in many studies. Here, indicate whether each material, system or method listed is relevant to your study. If you are not sure if a list item applies to your research, read the appropriate section before selecting a response.

Materials & experimental systems

n/a	Involved in the study	
[ ]	[x]	Antibodies
[ ]	[x]	Eukaryotic cell lines
[x]	[ ]	Palaeontology and archaeology
[ ]	[x]	Animals and other organisms
[x]	[ ]	Human research participants
[x]	[ ]	Clinical data
[x]	[ ]	Dual use research of concern

Methods

n/a	Involved in the study	
[x]	[ ]	ChIP-seq
[x]	[ ]	Flow cytometry
[x]	[ ]	MRI-based neuroimaging

Antibodies

Antibodies used	Primary antibodies<br>monoclonal mouse anti-Acetylated Tubulin (Sigma-Aldrich T7451) used at 1:400 dilution in the blocking buffer; a rabbit polyclonal anti-Laminin (Sigma L9393) used at 1:300; a rabbit polyclonal anti-Phospho-Histone H3 (Cell Signaling Technology 9701) used at 1:200; a mouse monoclonal anti-Tubulin beta3 (BioLegend 801201) used at 1:750; a rabbit polyclonal anti-doublecortin (Abcam, ab18723) used at 1:200; a rabbit monoclonal anti BRA (Abcam, ab209665) used at 1:1000; a rabbit polyclonal anti GFP (Novus Biologicals - NB600-308) used at 1:400; a rabbit polyclonal anti GATA6 (Invitrogen - PA1-104) used at 1:400; a rat monoclonal anti NANOG (Invitrogen -14-5761-80) used at 1:500; a rabbit polyclonal anti Sox2 (Millipore - ab5603) used at 1:500 and a goat polyclonal anti Sox17 (R&D Systems - AF1924) used at 1:20.<br>Secondary antibody: goat anti-rabbit Alexa Fluor 488 (Thermofisher, A11008) or a goat anti-mouse Alexa Fluor 546 (Thermofisher, A11030) used at a 1:800 dilution in the blocking buffer as well as a donkey anti mouse IGG Alexa Fluor 546 (Invitrogen A10036), a donkey anti rabbit IGG Alexa Fluor 546 (Invitrogen A10040), a donkey anti goat Alexa Fluor 488 (Jackson immuno research, 705-545-174) used at a 1:400 dilution in the blocking buffer

Validation	Each antibody was validated in the lab based on their tissue specific labelling of tissues and organs of mouse embryos at the appropriate developmental stages. Additional information on validation is available from websites of Millipore, Sigma, Cell Signaling Technology , BioLegend, Abcam, Novus Biologicals, Invitrogen

Eukaryotic cell lines

Policy information about cell lines

Cell line source(s)	mouse embryonic stem cell lines: Bra-GFP (G. Keller's lab, Fehling, H. J. et al. Tracking mesoderm induction and its specification to the hemangioblast during embryonic stem cell differentiation. Development 130, 4217-4227 (2003).). Sox1-GFP (A. Smith's lab, Aubert, J. et al. Screening for mammalian neural genes via fluorescence-activated cell sorter purification of neural precursors from Sox1-gfp knock-in mice. Proc. Natl. Acad. Sci. U S A 100 Suppl 1, 11836-11841 (2003)). E14TG2 line (non-labelled, wild-type cells, ATCC CRL-1821); and 129 ESC with GFP line (Cyagen MUAES-01101).
Authentication	Bra-GFP line is authenticated based on the expression of GFP in embryoid bodies treated in presence of BMP4 protein. Sox1-GFP line is authenticated based on the expression of the GFP in territories expressing the neural markers Sox1 and Sox2 in embryoids after D5; 129 ESC with GFP is authenticated based on the constitutive expression of the GFP by these cells. There is no known method to authenticate the unlabelled E14TG2 line.
Mycoplasma contamination	I confirm that all cell lines tested negative for mycoplasma contamination
Commonly misidentified lines (See ICLAC register)	There is no commonly misidentified lines used in this study.

Animals and other organisms

Policy information about studies involving animals; ARRIVE guidelines recommended for reporting animal research

Laboratory animals	Only fixed embryos from wild-type adult mice of various strains from Charles River Laboratories have been used in this study
Wild animals	N/A
Field-collected samples	N/A
Ethics oversight	N/A

Note that full information on the approval of the study protocol must also be provided in the manuscript.
