# Supplementary material for: Construction of a mammalian embryo model from stem cells organized by a morphogen signalling centre
Source: Nat Commun. 2021 Jun 2;12:3277. doi: 10.1038/s41467-021-23653-4 (PMC8172561; doi:10.1038/s41467-021-23653-4)
Supplement: Supplementary file 8 — Description of Additional Supplementary Files [file 41467_2021_23653_MOESM8_ESM.pdf]

Description of Additional Supplementary Files

File Name: Supplementary movie 1
Description: D6 embryoid imaged every 20 min for 16 hrs with 14 optical sections of 10 µm each using an HCX PL Fluotar L 20X/0.40 dry objective.

File Name: Supplementary movie 2
Description: D8 embryoid in lateral view imaged every 0.046 second for 12 seconds using an HC PL Fluotar 10X/0.30 dry objective.

File Name: Supplementary Data 1: Differentially Expressed Genes (DEG) markers for the 20 embryoid cell clusters.
Description: Table providing the list of differentially expressed gene markers for each cell cluster identified. p_val.: pvalue, pct1: percentage of cells where the gene is detected in the cluster, pct2: percentage of cells on average in the other clusters. p_val adj: Adjusted p-value, based on Bonferroni correction using all genes in the dataset, used to determine significance. Differential gene expression analysis was conducted between identified clusters to find marker genes for each cell type using Wilcoxon rank sum test (seurat default parameter).

File Name: Supplementary Data 2: Detailed information for the synthesis of antisense RNA probes Restriction enzyme: indicates the restriction endonuclease to be used to generate a linear DNA template.
Description :Primer 1 and Primer 2 are nucleotide sequences of the oligonucleotides to be used to generate a linear PCR fragment for the synthesis of the antisense RNA probe. Sequence column provide the Genebank accession number of the corresponding cDNA (when available). The RNA polymerase (either T3 RNA polymerase of T7 RNA polymerase) are the polymerases to be used for synthesis of the antisense RNA probe.
